# Wnt-beta-catenin pathway signals metastasis-associated tumor cell phenotypes in triple negative breast cancers

**DOI:** 10.18632/oncotarget.8988

**Published:** 2016-04-26

**Authors:** Pradip De, Jennifer H. Carlson, Hui Wu, Adam Marcus, Brian Leyland-Jones, Nandini Dey

**Affiliations:** ^1^ Department of Molecular & Experimental Medicine, Avera Research Institute, Sioux Falls, SD, USA; ^2^ Department of Internal Medicine, SSOM, University of South Dakota, Sioux Falls, SD, USA; ^3^ Department of Hematology and Oncology, WCI, Emory University, Atlanta, GA, USA

**Keywords:** integrin-directed migration, matrigel-invasion, Wnt-pathway modulators, LWnt3ACM stimulation, brain-metastasis specific TNBC cells

## Abstract

Tumor cells acquire metastasis-associated (MA) phenotypes following genetic alterations in them which cause deregulation of different signaling pathways. Earlier, we reported that an upregulation of the Wnt-beta-catenin pathway (WP) is one of the genetic salient features of triple-negative breast cancer (TNBC), and WP signaling is associated with metastasis in TNBC. Using cBioPortal, here we found that collective % of alteration(s) in WP genes, CTNNB1, APC and DVL1 among breast-invasive-carcinomas was 21% as compared to 56% in PAM50 Basal. To understand the functional relevance of WP in the biology of heterogeneous/metastasizing TNBC cells, we undertook this comprehensive study using 15 cell lines in which we examined the role of WP in the context of integrin-dependent MA-phenotypes. Directional movement of tumor cells was observed by confocal immunofluorescence microscopy and quantitative confocal-video-microscopy while matrigel-invasion was studied by MMP7-specific casein-zymography. WntC59, XAV939, sulindac sulfide and beta-catenin siRNA (1) inhibited fibronectin-directed migration, (2) decreased podia-parameters and motility-descriptors, (3) altered filamentous-actin, (4) decreased matrigel-invasion and (5) inhibited cell proliferation as well as 3D clonogenic growth. Sulindac sulfide and beta-catenin siRNA decreased beta-catenin/active-beta-catenin and MMP7. LWnt3ACM-stimulated proliferation, clonogenicity, fibronection-directed migration and matrigel-invasion were perturbed by WP-modulators, sulindac sulfide and GDC-0941. We studied a direct involvement of WP in metastasis by stimulating brain-metastasis-specific MDA-MB231BR cells to demonstrate that LWnt3ACM-stimulated proliferation, clonogenicity and migration were blocked following sulindac sulfide, GDC-0941 and beta-catenin knockdown. We present the first evidence showing a direct functional relationship between WP activation and integrin-dependent MA-phenotypes. By proving the functional relationship between WP activation and MA-phenotypes, our data mechanistically explains (1) why different components of WP are upregulated in TNBC, (2) how WP activation is associated with metastasis and (3) how integrin-dependent MA-phenotypes can be regulated by mitigating the WP.

## INTRODUCTION

The triple negative (TN) subtype of breast cancers (BC) represents 15-20% of breast tumors (BT) which are more commonly diagnosed in younger African American women with the prevalence of BRCA1/2 mutations [1–3]. TNBC subtype is the most challenging diagnosis among BC patients due to the aggressive nature of the disease and the high metastatic potency of tumor cells which is responsible for the dismal clinical outcome as compared to other BC subtypes [4]. Therapeutic options for patients with TNBC are limited because no pathway-specific targets and associated biomarkers have been established [5–7]. Such a situation is further challenged by the fact that the disease is heterogeneous [8–10]. Although reports have indicated the involvement of certain genes/signaling molecules related to tumorigenic/metastatic pathways [5, 11–13] [14] in this subset of BC, there remains an unmet need for an in-depth study to establish a pathway-specific targeted therapy in TNBC, especially in the context of metastatic setting [15].

The Wnt-beta-catenin pathway (WP) is a ligand-driven signaling pathway which regulates several cellular phenotypes in development and disease [16, 17]. Activation of the pathway leads to a context-dependent transcription of beta-catenin target genes including MMP7 and c-MYC to directly control cellular phenotypes including survival, proliferation, migration, polarity and matrix remodeling [18].

We and others have demonstrated a subtype-specific association of the WP upregulation in BC. Reis-Filho's team reported that WP activation is (1) preferentially found in TN/basal-like breast carcinomas, (2) associated with poor clinical outcome and (3) unlikely to be driven by CTNNB1 mutations in BC [19]. In line with this report, we have demonstrated a differential upregulation of the various components of the WP at both transcript and protein levels (in tumor samples from multiple cohorts and cell line based models) in this subset of BC [20]. Our data are in agreement with a previous report by Khramtsov *et al.*, regarding an association of WP signaling in poor-prognosis TNBC patients with metastasis [21]. In line with the above results, we have identified that the differential upregulation of WP in TNBC is associated with poor prognosis and metastasis [20].

Although all the above studies collectively identify that WP upregulation is one of the salient genetic features of TNBC, the role of the WP in the control of tumorigenic signals and MA phenotypes in TNBC cells is not known yet. Since the outcome of TNBC is connected to the aggressive metastatic behavior of the disease and a differentially upregulated WP in this subtype is associated with poor prognosis and metastasis [20], we hypothesized that the upregulation of WP in aggressive TNBC is functionally associated with metastasis-associated (MA) phenotypes. Thus to understand the functional relevance of the WP pathway in the biology of metastasizing TNBC tumor cells, we have undertaken a comprehensive and in-depth study in which the involvement of WP is tested in the context of MA phenotypes.

Here we demonstrated that the WP is causally associated with the regulation of key components of integrin-mediated MA phenotypes including fibronectin-directed migration, matrigel-invasion and clonogenicity in TNBC. Considering the fact that a significant percentage of the patients with TNBC develop brain metastasis [22], we extended our study towards testing the role of WP in the metastatic settings by using brain-metastasis specific TNBC cells (the brain-tropic 231-BR experimental brain metastasis model of TNBC) [23], MDA-MB231BR and demonstrated that LWnt3ACM stimulated cell proliferation, clonogenicity and fibronectin-directed migration were abrogated in MDA-MB231BR cells by the blockade of WP signals following the treatment with sulindac sulfide, WP modulators, GDC-0941 and siRNA, which downregulated beta-catenin. Based on our [24] and others’ reports [19, 21] that WP is differentially activated in TNBC, we here establish the function of WP in TNBC especially in metastatic settings to mechanistically explain our previous observation that Wnt signaling in TNBC is associated with metastasis [20] which leads to poor outcome [21]. In the light of (1) the report from Reis-Filho's team demonstrating a preferential increase in beta-catenin protein (IHC) in TNBC patients, (2) *CTNNB1* amplification observed in tumors from our Avera patients and (3) our previous report, here we present the first genetic and pharmacological evidence to demonstrate a direct functional relationship between the activation of the WP and key components of MA phenotypes. To the best our knowledge, this is the first report to reveal a direct functional connection between subset-specific upregulation of the WP and key components of integrin-mediated MA phenotypes in TNBC more specifically in the context of brain metastasis.

## RESULTS

### Alterations of *CTNNB1*, *APC* and *DVL1* genes in all BC cases and different BC subtypes, cBioPortal data

**Figure 1:**
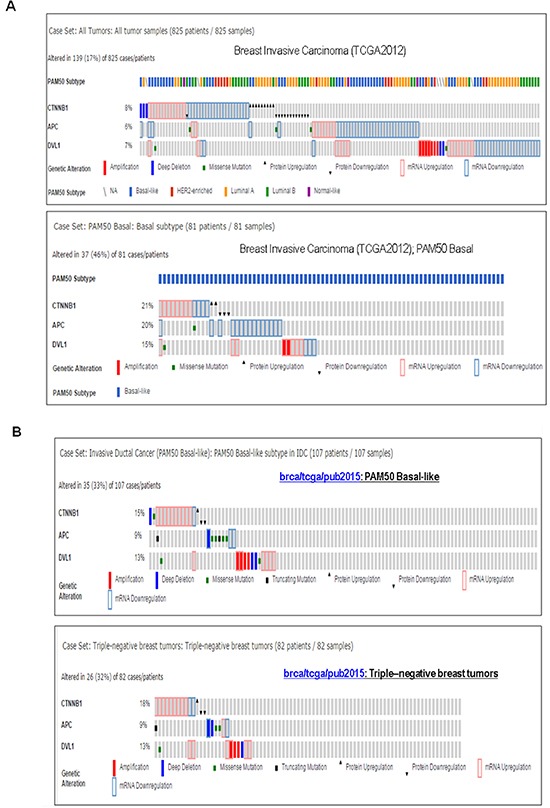
Alterations of WP genes in TNBC and basal-like BC subtypes **A.** Oncoprints showing alterations in WP associated *CTNNB1, APC and DVL1* in Breast Invasive Carcinoma (upper panel) and Breast Invasive Carcinoma; PAM50 Basal-like (lower panel). The patient selected were, (1) Breast Invasive Carcinoma; TCGA 2012 (825 patients/825 samples), and (2) Breast Invasive Carcinoma (TCGA 2012); PAM50 Basal (81 patients/81 samples). **B.** Oncoprints showing alterations in *CTNNB1, APC and DVL1* in PAM50 Basal-like brca/tcga/pub2015 (upper panel) and triple-negative breast tumors brca/tcga/pub2015 (lower panel). The oncoprints are generated using 107 patients/107 samples for PAM50 Basal-like and 82 patients/82 samples for Triple-negative breast tumors. Advanced cancer genomic data visualization is obtained with the help of “The Onco Query Language (OQL)”. Oncoprints (different levels of zoom) have been generated using cBioPortal. Individual genes are represented as rows, and individual cases or patients are represented as columns. Protein level obtained from IHC staining (cBioPortal).

Percentages of alterations in the WP specific *CTNNB1, APC* and *DVL1* genes among all tumors samples of breast invasive carcinomas (TCGA 2012) varied from 6-8% in between individual genes (*CTNNB1*, 8%; *APC*, 6%; *DVL1,* 7%) while alterations of same genes among breast invasive carcinomas, PAM50 Basal-like (TCGA 2012) varied from 15-21% (*CTNNB1*, 21%; *APC,* 20%; *DVL1*, 15%). The collective % of alterations in *CTNNB1, APC* and *DVL1* genes among total breast invasive carcinomas (TCGA 2012) were 21% in contrast to 56% breast invasive carcinomas, PAM50 Basal-like (TCGA 2012) (Figure 1A). A similar trend was observed among subtypes of tumors from brca/tcga/pub2015 (cBioPortal). In this data set, the percentage of alterations in *CTNNB1*, *APC* and *DVL1* genes among all tumors samples (1105 cases/patients) varied from 5-8% in between individual genes (*CTNNB1*, 8%; *APC*, 7%; *DVL1,* 5%). The collective percentage of alterations in *CTNNB1, APC* and *DVL1* genes among total 1105 cases/patients were 20%. Although the collective changes in the percentage varied between luminal A (8%), luminal B (17%) and Her2-enriched (26%) subtypes, the pattern of percentage changes of the individual genes of *CTNNB1, APC* and *DVL1* in luminal A, luminal B and Her2-enriched subtypes remained comparable to TCGA2012 data set (Supplementary Figure S5). In contrast, both collective changes in the percentage (37% in PAM50 Basal-like subtype of IDC and 40% in triple negative breast tumors) as well as the percentage of alterations of individual genes of *CTNNB1* (15% in PAM50 Basal-like subtype of IDC and 18% in triple negative breast tumors), *APC* (9% for both) and *DVL1* (13% for both) were found significantly higher in both PAM50 Basal-like subtype of IDC and triple negative breast tumors as compared to other subtypes of BC (Figure 1B).

### WP signaling inhibitor, sulindac sulfide downregulated total beta-catenin levels in MDA-MB468 and Hs578t TNBC cells

**Figure 2:**
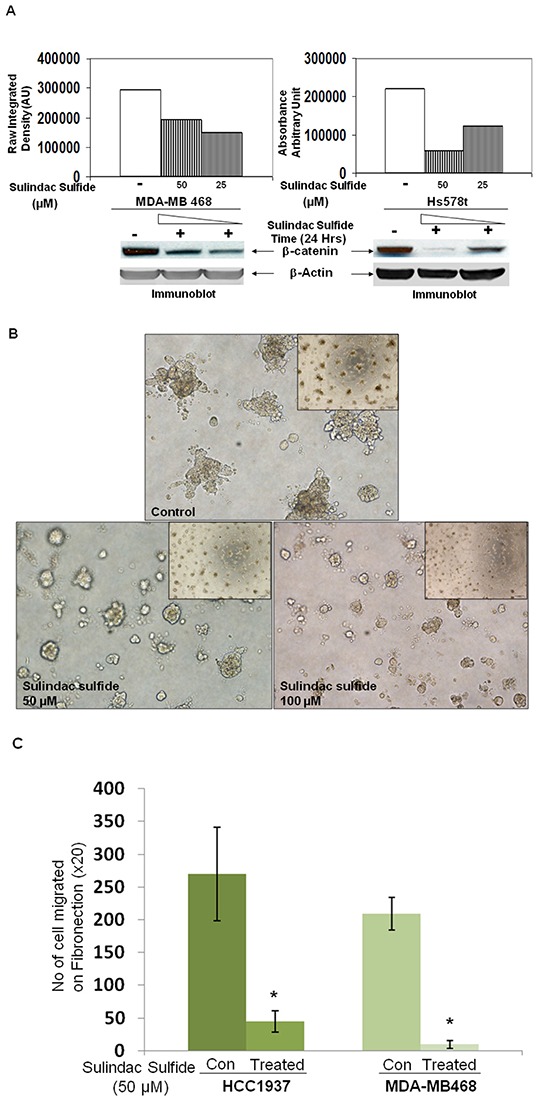

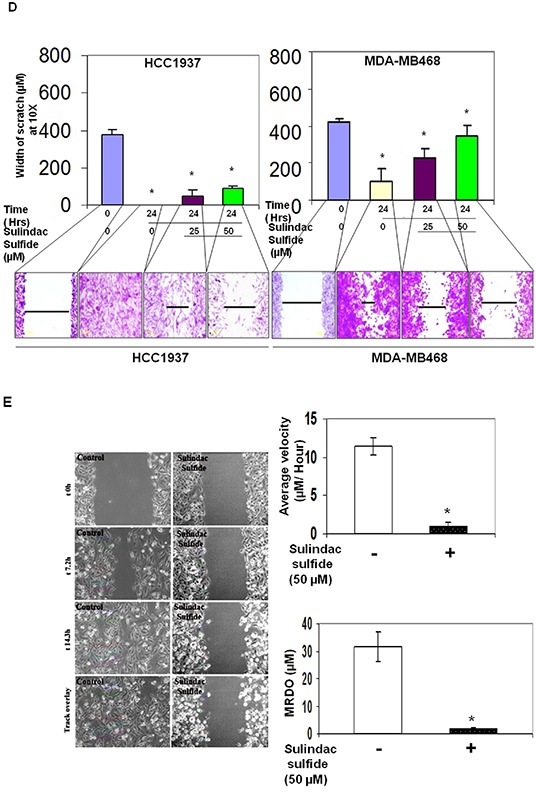
Effect of sulindac sulfide on (A) the expression of total beta-catenin protein, (B) the clonogenic 3D growth, (C) fibronectin-mediated migration by transwell assay, (D) fibronectin-mediated migration by scratch assay and (E) motility descriptors of real-time movement of live TNBC cells **A.** Cells were treated with sulindac sulfide (25 μM and 50 μM) and total beta-catenin levels were determined by WB. **B.** Cells were treated with sulindac sulfide (25 μM and 50 μM) and their 3D clonogenic growth was tested. **C.** Sulindac sulfide treatment blocked fibronectin-mediated migration of HCC1937 and MDA-MB468 in a transwell assay (*p< 0.05). **D.** Sulindac sulfide treatment dose-dependently (25 μM and 50 μM) inhibited migration of HCC1937 and MDA-MB468 TNBC cells in the scratch assay (*p< 0.05). **E.** Sulindac sulfide (50 μM) also blocked (*p< 0.05) the trajectory of the movement of MDA-MB231 cells as measured by the nuclear tracking paths of the migrating live cells semi-quantified by two motility descriptors, average velocity (Upper bar diagram on the left) and MRDO, maximum relative distance from the origin (Upper bar diagram on the right). Vehicle-treated (control) and sulindac sulfide treated cells are imaged in real time. Nuclear tracking paths of the migrating cells are shown as track overlays. Bars represent Mean ± S.D. of the average velocity (μM/Hour) and MRDO (μM) of the cells in the presence (dark vertical bar) and absence (open bar) of sulindac sulfide. *p<0.01.

Ligand-receptor engagement in the WP has been known to increase the half-life of beta-catenin (Figure 10) by blocking the degradation of beta-catenin. According to the model described by Staal et al., the changes in beta-catenin stability set the threshold of Wnt signaling [28]. We used WP signaling inhibitor, sulindac sulfide to downregulate cellular levels of beta-catenin in some TNBC cell lines. Our *in vitro* phenotypic experiments focused on beta-catenin because beta-catenin is the functional as well as a biochemical readout of WP and it can be pharmacologically targeted (by sulindac sulfide) as well as tested in clinical trials [29, 30]. Sulindac and its derivatives are known to decrease beta-catenin expression and beta-catenin transcriptional activities in breast cancer and APC-mutated colorectal tumor cells [31–33]. Sulindac sulfide treatment dose-dependently decreased total beta-catenin levels in MDA-MB468 and Hs578t TNBC cells (Figure 2A). Results have been semi-quantified using Image J. In our previous studies we also demonstrated that sulindac sulfide administration (25-100 μM) caused a dose-dependent decrease in cellular levels of beta-catenin in BT20 and SUM149 TNBC cells [20, 24, 34]. A similar dose and time-dependent attenuation of beta-catenin levels were also reported as early as 24 hours after treatment with sulindac sulfide or sulindac sulfone (ranges 120-600μM) via three different ways of induction of beta-catenin degradation [35]. We observed a similar decrease in the levels of beta-catenin in MDA-MB468 and Hs578t (Figure 2A) as well as BT20 and SUM149 TNBC cells [20, 24] which justified our use of sulindac sulfide in the subsequent experiments.

### Effects of sulindac sulfide on metastasis-associated phenotypes in TNBC cells

Having used sulindac sulfide to decrease cellular levels of beta-catenin, we went on to test the effect of the inhibitor on different phenotypes. They are (i) 3D clonogenic growth, (ii) fibronectin-directed migration in transwell and scratch assays, (iii) fibronectin-directed real-time movement of live cells in wound-healing assay, (iv) motility descriptors including average velocity (AV) and maximum relative distance from the origin (MRDO) in live cells, (v) architecture of filamentous actin on fibronectin, (vi) podia-parameters (filopodia and lamellipodia), (vii) fibronectin-directed matrigel-invasion and (viii) levels of active beta-catenin as well as the expression of its invasion specific target gene, MMP7. Sulindac sulfide had been known to inhibit the proliferation of colon cancer cells and diminished expression of the proliferation markers PCNA and Ki-67 [36]. We first tested the effect of sulindac sulfide on the clonogenic growth of TNBC cells. Sulindac sulfide dose-dependently (50 and 100 microM) blocked the 3D clonogenic growth in SUM149 cells (Figure 2B).

Sulindac sulfide blocked fibronectin-directed transwell migration in HCC1937 and MDA-MB468 cells (Figure 2C; Supplementary Figure S4) as well as abrogated fibronectin-directed migration (scratch assay) (Figure 2D). Supplementary Figure S2 shows the migratory property (transwell and scratch assays) of different BC cell lines. Next, we tested the fibronectin-directed real-time movement of live MDA-MB231 cells in wound-healing assay following sulindac sulfide treatment (Supplementary Movie: M1 & M2). As sulindac sulfide inhibited fibronectin-directed real-time movement of live MDA-MB231 cells in a wound-healing assay, we tested the effect of the drug on motility descriptors, average velocity (AV) and maximum relative distance from the origin (MRDO) in live MDA-MB231 cells (Figure 2E). Vehicle-treated (control) and sulindac sulfide treated (50 microM) cells are imaged for the purpose. Image analysis from the time-lapse images of fibronectin-directed migration of cells in scratch wound healing assay demonstrated that sulindac sulfide (50 microM) administration impaired migration of cells. Control cells migrated exhibiting directional movement into the fibronectin coated area (open space) with well-formed lamellae protruding from the leading edge of cells (Figure 2E). In contrast, treated cells exhibited truncated lamellae in limited numbers which corresponded with their limited migration. Nuclear tracking paths of the migrating cells as shown in track overlays demonstrated the trajectory of the movement of the cells characterized by two quantitative motility descriptors, AV and MRDO. Data showed that the treatment of sulindac sulfide has a significant anti-migratory effect on TNBC cells.

**Figure 3:**
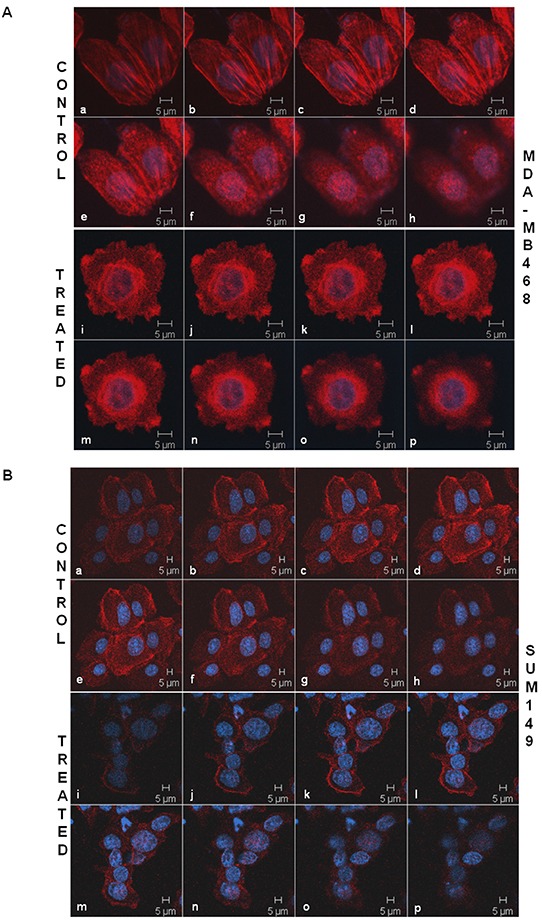
Sulindac sulfide treatment caused a loss of the cytoskeletal architecture of the filamentous actin in MDA-MB468 A. and SUM149 B. cells TNBC cells were stained with phalloidin 555 for the confocal microscopy to test the effect of sulindac sulfide on the cytoskeletal architecture of the filamentous actin. The cells were stained with DAPI as a counterstain. Cells are imaged using Zeiss LSM 510 Metasystem. Successive Z-sections across cells were represented in the picture (consecutive photomicrographs) to demonstrate the effect of sulindac sulfide (photomicrographs i-p) on the organization of the filamentous actin as compared to the respective controls (photomicrographs a-h).

**Figure 4:**
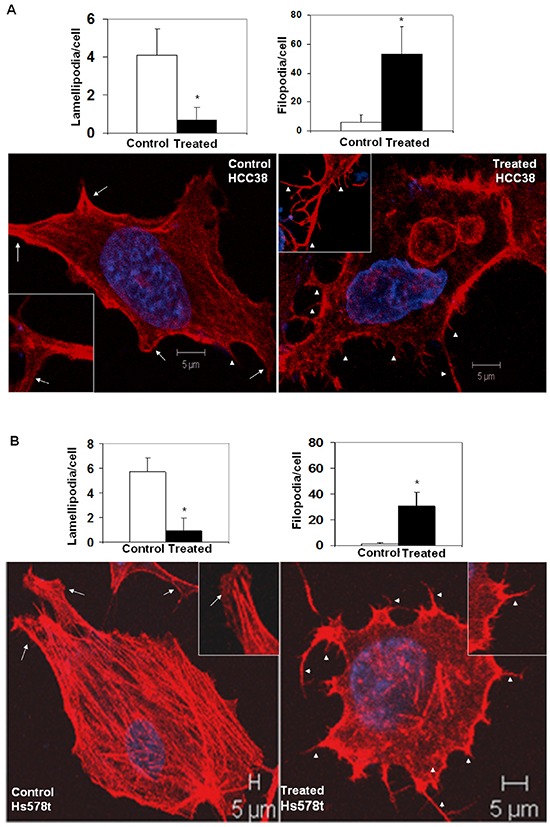
Sulindac sulfide treatment altered podia-parameters (lamellipodia and filopodia) of HCC38 A. and Hs578t B. cells TNBC cells were cultured on fibronectin-coated coverslips. Control and sulindac sulfide treated (50 μM) cells were fixed in warm reconstituted PHEMO buffer before they were stained with phalloidin 555 and counterstained with DAPI. Filopodia (short arrowheads) and lamellipodia (arrows) were identified under a confocal microscope (Z-sections) as presented in the inset of individual cells. The semi-quantification was performed using ten randomly chosen fields from 4 independently performed experiments (*p<0.01).

The intracellular architecture of filamentous actin directly controls the integrin-guided movements of cells. Since we observed blockade of cell movement in real time and demonstrated the abrogation of the motility descriptors following sulindac sulfide in TNBC cells, we wanted to test the effect of the drug on the architecture of filamentous actin. Figure 3 showed that the treatment with sulindac sulfide disrupted the architecture of filamentous actin in MDA-MB468 and SUM149 TNBC cells (Figure 3) on fibronectin. The formation of lamellipodia and filopodia are directly controlled by the intracellular architecture of filamentous actin. Hence, we next tested the podia-parameters of TNBC cells on fibronectin following the treatment of cells with sulindac sulfide. Figure 4 showed that sulindac sulfide decreased lamellipodia of HCC38 and Hs578t TNBC cells on fibronectin while the filopodia numbers per cell were increased.

**Figure 5:**
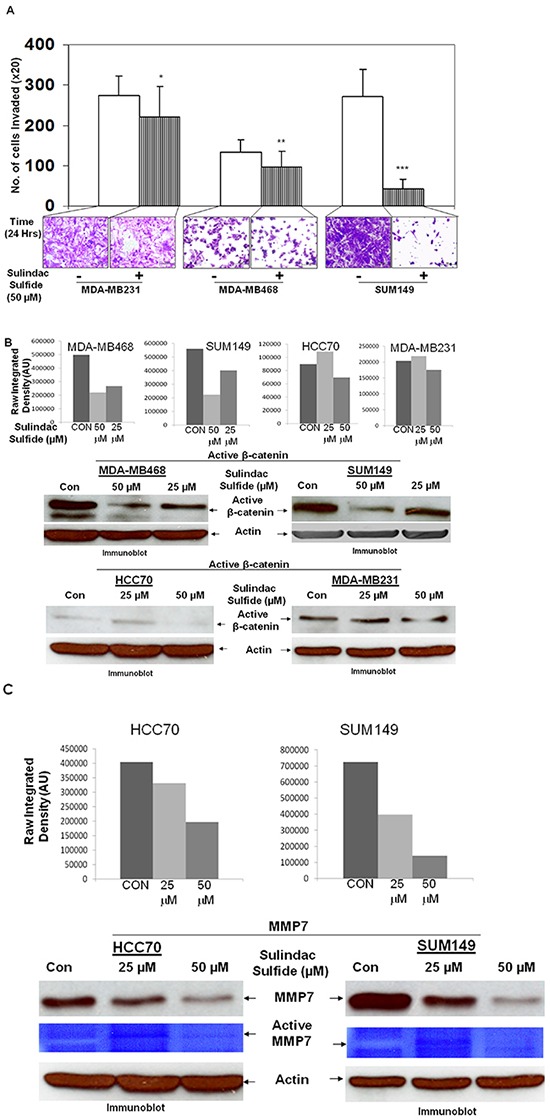
Effect of Sulindac sulfide treatment on fibronectin-mediated invasion (A), levels of active beta-catenin (B) and expression/function of MMP7, a transcriptional target of active beta-catenin (C) in different TNBC cells **A.** Sulindac sulfide (50 μM) blocked fibronectin-mediated invasion of MDA-MB231, MDA-MB468 and SUM149 TNBC cells through matrigel in the transwell invasion assay (**p< 0.05; ***p< 0.0001; *p< 0.07). **B.** Sulindac sulfide dose-dependently (25 μM and 50 μM) blocked the transcriptionally active form of beta-catenin in MDA-MB468, SUM149, HCC70 and MDA-MB231 TNBC cells. The expression of active beta-catenin was semi-quantified using ImageJ. **C.** Sulindac sulfide dose-dependently (25 μM and 50 μM) decreased both the levels of the MMP7 protein as well as its function (casein zymogram from secreted MMP7 in the conditioned media) in HCC70 and SUM149 TNBC cells. The expression of MMP7 protein was semi-quantified using ImageJ.

Integrin-directed migration of tumor cells helps them to invade through ECM. We studied fibronectin-directed matrigel-invasion as one of the MA phenotypes because it involves migration of cells as well as requires matrix degradation activity of matrix metalloprotease-like MMP7 which is a WP specific ECM-degrading metalloprotease [24]. Figure 5 showed that sulindac sulfide blocked fibronectin-directed matrigel-invasion of MDA-MB231, MDA-MB468 and SUM149 cells (Figure 5A). We observed that different TNBC cells differed in their capacity to invade matrigel. However, the inhibitory effect of sulindac sulfide was observed in all cell lines tested. Dephosphorylated beta-catenin is the transcriptionally active form because it moves to the nucleus and is competent of initiating the transcription of its target genes [37]. Upon activation of the WP, degradation of active cytosolic beta-catenin is blocked which then enters the nucleus to initiate the transcription of its target genes including MMP7 (Figure 10). We reported that differential activation of WP in TNBC increases MMP7 [24], a target gene of WP involved in the degradation of matrix protein during the invasion. Hence to provide mechanistic insight into the mode of involvement of WP in fibronectin-directed matrigel invasion, we studied the effect of sulindac sulfide on the levels of active beta-catenin as well as the expression of MMP7 in TNBC cells. Sulindac sulfide treatment dose-dependently blocked active beta-catenin (Figure 5B) and decreased both the expression and activity of MMP7 (Figure 5C) in TNBC cells. The decreased levels of active beta-catenin reciprocated with the downregulation of MMP7 levels/function (casein-zymography) in HCC70 and SUM149 cells. This decrease in the MMP7 level in SUM149 cells is in line with our previously reported loss of MMP7 casein-zymographic activity as well as the relative luciferase activity (TOP-Flash over FOP-Flash) following sulindac sulfide treatment [24]. Results show that sulindac sulfide treatment blocked the fibronectin-mediated invasion of TNBC cells via direct inhibition of the transcriptional target of active beta-catenin, MMP7 in cells which endogenously express MMP7. Interestingly, the effect of sulindac sulfide on the invasion of MDA-MB231 cells which do not express the measurable amount of MMP7 reflects the effect of sulindac sulfide on the migratory property of the cell. Using ApcMin/+ mice, a genetic model of human familial adenomatous polyposis, Guillen-Ahlers et al., reported a downregulation of MMP7 vis-à-vis reduction of tumors in sulindac-treated animals which was further validated by RT-PCR. MMP7 in their study as found in ‘hot spot’ areas within the tumors of vehicle-treated animals in contrast, was greatly diminished in those mice treated with sulindac [38]. Data represented in Figure 5 also substantiate our previous report that the relative luciferase activity (TOP-Flash over FOP-Flash) in MDA-MB231 cells were decreased following downregulation of sulindac sulfide-mediated beta-catenin and active beta-catenin levels [20]. We did not perform MMP7 assay in MDA-MB231 as we previously observed that the MMP7 protein expression and its enzymatic activity (by casein zymography) is negligible in MDA-MB231 cells [24].

### Physiological stimulation of WP & Effect of WP inhibition on LWnt3ACM-stimulated metastasis-associated phenotypes in TNBC cells

In addition to the perturbation of the WP by sulindac sulfide, we also stimulated (ligand-mediated) the WP by LWnt3ACM. Treatment of cells with LWnt3ACM increased cellular levels of beta-catenin (data not shown). To demonstrate that WP is causally responsible for the regulation of MA phenotypes in TNBC, cells were physiologically stimulated with LWnt3ACM and the effect of pharmacological modulators of different nodes of the WP (CHIR99021, WntC59, XAV939 and GDC-0941) was studied.

**Figure 6:**
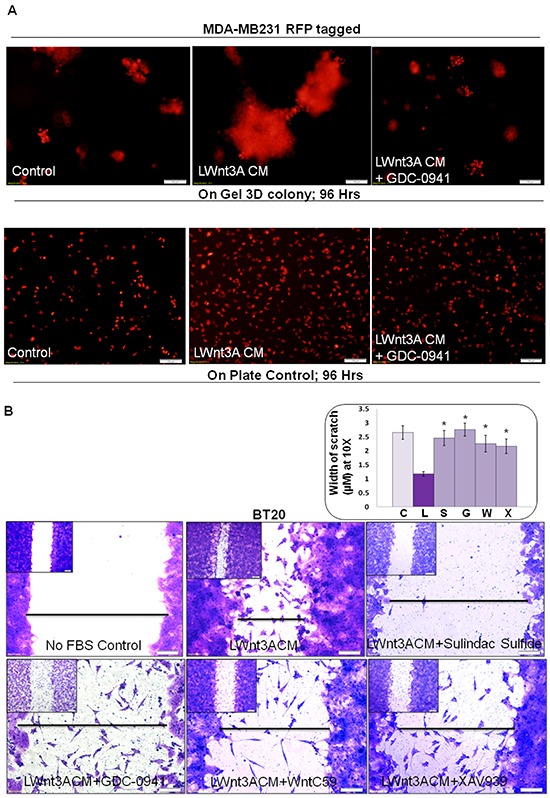
Effect of pan-PI3K inhibitor GDC-0941, sulindac sulfide, WntC59 and XAV939 on LWnt3ACM stimulated 3D-ON-TOP growth in MDA-MB231Red (A) and fibronectin-directed migration in BT20 (B) cells **A.** GDC-0941 (1 μM) blocked LWnt3ACM stimulated 3D-ON-TOP growth at 96 hours in MDA-MB231Red TNBC cells. The “on plate” controls were included as the internal control to show the culture condition of the survival of the cells at 96 hours as the experiments were conducted under no FBS condition. The experiments were conducted under FBS-free condition since the LWnt3ACM was collected under no FBS condition. **B.** Sulindac sulfide, GDC-0941, WntC59 and XAV939 abrogated LWnt3ACM stimulated fibronectin-directed migration at 24 hours in BT20 cells. Low magnification pictures were presented as insets. The distance between scratches (n=4) was semi-quantified from 10 randomly chosen fields and presented as bar-diagram where FBS-free control (C) was compared with LWnt3ACM stimulated (L). The increase in the migration in the LWnt3ACM stimulated cells (as measured by the decrease in the width of the scratches) was compared with LWnt3ACM stimulated plus sulindac sulfide (S), LWnt3ACM stimulated plus GDC-0941(G), LWnt3ACM stimulated plus WntC59 (W), LWnt3ACM stimulated plus XAV939 (X) (*p< 0.05).

Upregulation of the PI3K-AKT pathway decreases degradation of beta-catenin via inhibition of GSK3beta (as a part of beta-catenin degradation complex). We have previously reported that pan PI3K inhibitor LY294002, as well as SF1126, blocked phosphorylation of GSK3beta in HCC1937, MDA-MB231 and MDA-MB468 TNBC cells [34]. In agreement with the above fact, we reported that the treatment with LY294002 decreased both cellular levels of MMP7 as well as enzymatic activity (casein zymography) of MMP7 in TNBC cells [24] indicating that inhibition of the PI3K-AKT pathway blocks WP signals. Accordingly, here we tested the effect of the pan-PI3K inhibitor on the LWnt3ACM-stimulated 3D clonogenic growth of TNBC cells. Figure 6A shows that LWnt3ACM-stimulated 3D clonogenic growth (96 hrs) was blocked by the pan-PI3K inhibitor, GDC-0941. We also stimulated fibronectin-directed transwell migration using LWnt3ACM and tested the effect of some WP modulating agents including sulindac sulfide, GDC-0941, WntC59 and XAV939. Figure 6B showed that LWnt3ACM-stimulated fibronectin-directed migration in TNBC cells was inhibited in the presence of WP signaling inhibitor, sulindac sulfide and GDC-0941 as well as WP modulators like WntC59 and XAV939 in BT20 cells. Since we stimulated the WP by Wnt3A, we included different inhibitors that block WP signaling from different nodes. Data showed that the downregulation of WP by, (1) decreasing receptor-ligand interaction following WntC59 (prevents palmitoylation of Wnt proteins by Porcupine, a membrane-bound O-acyltransferase and thereby blocks Wnt secretion and activity), (2) stabilizing Axin1 and Axin2 protein levels following XAV939 (a small molecule tankyrase inhibitor which enhances the degradation of the beta-catenin and attenuates Wnt-induced transcriptional activity of beta-catenin), (3) WP signaling inhibitor, sulindac sulfide and (4) PI3K-AKT-GSK3beta inhibition following GDC-0941(reduces beta-catenin levels) blocked LWnt3ACM-stimulated migration of TNBC cells on fibronectin.

### Effect of beta-catenin siRNA-mediated inhibition of WP on metastasis-associated phenotypes in TNBC

**Figure 7:**
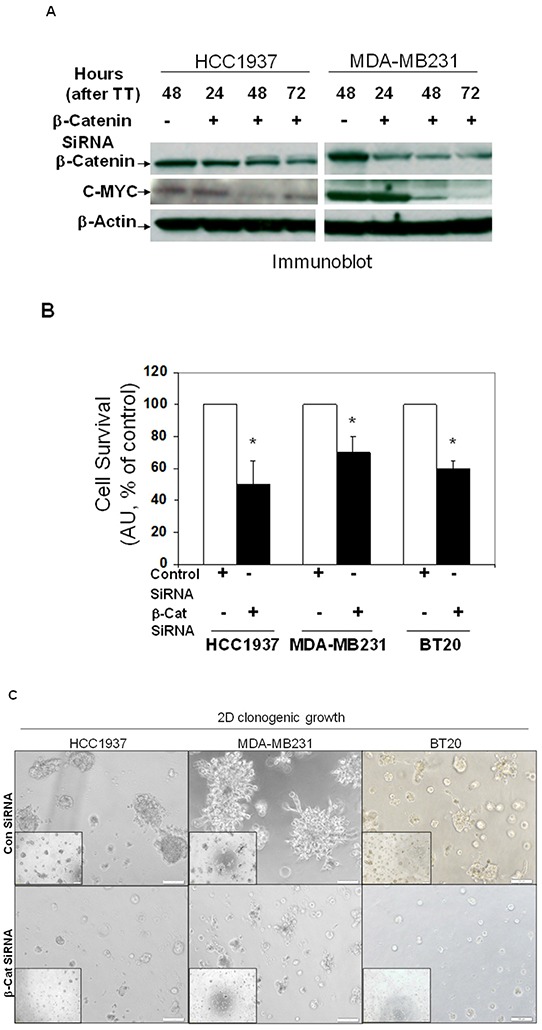

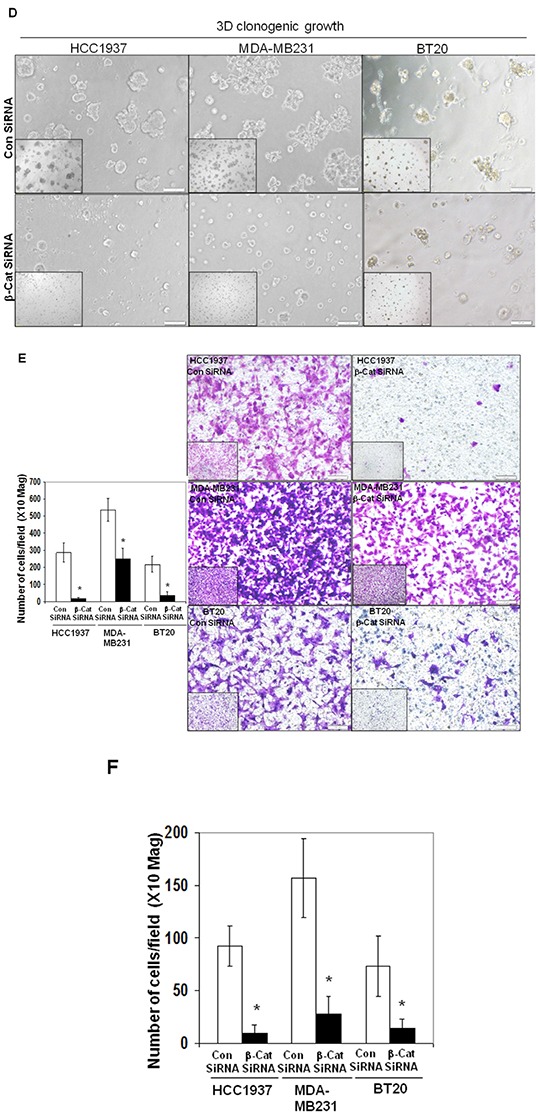
SiRNA mediated downregulation of beta-catenin and c-MYC proteins in TNBC cells (A) caused a decreased cell survival (B), blocked clonogenic 2D growth (C), blocked 3D ON-TOP growth. (D), inhibited fibronectin-directed transwell migration (E) and inhibited matrigel invasion (F) **A.** HCC1937 and MDA-MB231 cells were transiently transfected with beta-catenin siRNA and control siRNA for different time points. Actin was used as the loading control. c-MYC levels were determined after transient transfection of beta-catenin siRNA. Downregulation of beta-catenin was tested as the reference. **B.** Effect of downregulation of beta-catenin was tested on the proliferation of HCC1937, MDA-MB231 and BT20 cells by cell TiterGLO following transient transfection of beta-catenin siRNA for 72 hours. Data presented as % of the live cell (*p< 0.05). **C.** Effect of downregulation of beta-catenin following transient transfection of beta-catenin siRNA was tested on the 2D clonogenic growth of HCC1937, MDA-MB231 and BT20 cells. **D.** Effect of downregulation of beta-catenin following transient transfection of beta-catenin siRNA was tested on the 3D clonogenic growth of HCC1937, MDA-MB231 and BT20 cells. Cells were plated for the clonogenic assay 24 hours following the transfection and clonogenicity were tested for 72 hours. **E.** Effect of downregulation of beta-catenin following transient transfection of beta-catenin siRNA (for 24 hours) was tested on the fibronectin-directed migration of HCC1937, MDA-MB231 and BT20 cells in a transwell. Migrated cells were stained with crystal violet before counting under a microscope (10 random fields) (*p< 0.05). Low-magnification images were presented as insets. **F.** Effect of siRNA-mediated down regulation of beta-catenin on matrigel invasion was tested in HCC1937, MDA-MB231, and BT20 cells following transient transfection of beta-catenin siRNA for 24 hours. Invaded cells were stained with crystal violet before counting under a microscope (10 random fields) (*p< 0.05).

Beta-catenin is the functional readout of WP and we have reported the functional perturbation of the WP following transient transfection of beta-catenin siRNA [34]. We reported that transfection of beta-catenin siRNA decreased levels of total beta-catenin, active beta-catenin, relative luciferase activity (TOP-Flash), total protein levels of MMP7 and functional levels of MMP7 (casein zymogram) [20, 24]. Figure 7A showed the time course of siRNA-mediated depletion of beta-catenin in HCC1937 and MDA-MB231 cells. The control lysates were obtained at the mid-point (48 hrs) of the transfection. The efficiency of the transient transfection was tested in BT20, MDA-MB231 and HCC1937 cells (Supplementary Figure S1). To test the downstream read out of the decrease of total beta-catenin levels, we also determined the effect of siRNA-mediated downregulation of beta-catenin on one of the beta-catenin target genes, *c-MYC*. Figure 7A showed that c-MYC protein levels were decreased at both 48 and 72 hours after transfection of the siRNA as compared to their respective controls in both HCC1937 and MDA-MB231 TNBC cells confirming that the siRNA-mediated decrease in the level of beta-catenin led to the transcriptional downregulation of the WP. We next tested the effect of downregulation of beta-catenin by siRNA on the survival of HCC1937, MDA-MB231 and BT20 cells by cell TiterGLO (Figure 7B) (on the percentage of live cells) at 72 hours of transfection. Data showed that the survival of cells was decreased by 50% in HCC1937 cells transfected with beta-catenin siRNA as compared to the control siRNA. Similarly, 35% and 40% decrease in cell survival were observed in MDA-MB231 and BT20 cells respectively. SiRNA mediated downregulation of WP blocked both 2D (Figure 7C) and 3D (Figure 7D) clonogenic growth of TNBC cells as compared to their respective controls. The beta-catenin siRNA transfected HCC1937, MDA-MB231 and BT20 cells migrated significantly in lesser numbers as compared to their corresponding control siRNA-transfected cells (Figure 7E) indicating that siRNA-mediated downregulation of WP blocked fibronectin-mediated migration of TNBC cells. It is worth noting that the effect of siRNA-mediated downregulation of WP on the fibronectin-mediated migration was most pronounced in *PIK3CA* mutated BT20 cells and *PTEN* null HCC1937 cells as compared to the *RAS/RAF* mutated MDA-MB231 cells. We next tested the effect of beta-catenin siRNA-mediated downregulation of WP on matrigel invasion in these cells. Figure 7F showed the abrogation of fibronectin-mediated matrigel invasion following transfection of beta-catenin siRNA. Interestingly enough we observed a similarity in the pattern of inhibition of the invasion of cells with that of the migration of the cells which may indicate the distinct role of migration on the invasion of a tumor cell. Since none of the HCC1937, MDA-MB231 and BT20 cells expressed MMP7 (in contrast to that in SUM149) as we reported previously [24], it is interesting to note that the effect of beta-catenin siRNA on the invasion of these cells may be mediated directly via migration and not via MMP7.

### Effect of pharmacological modulators of different nodes of WP on metastasis-associated phenotypes in TNBC

**Figure 8:**
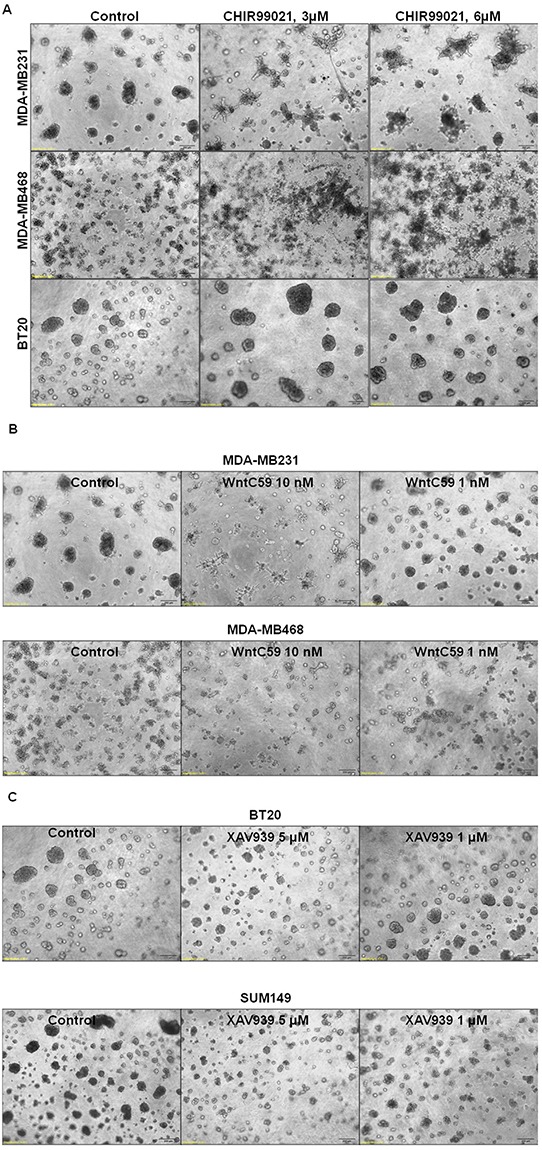

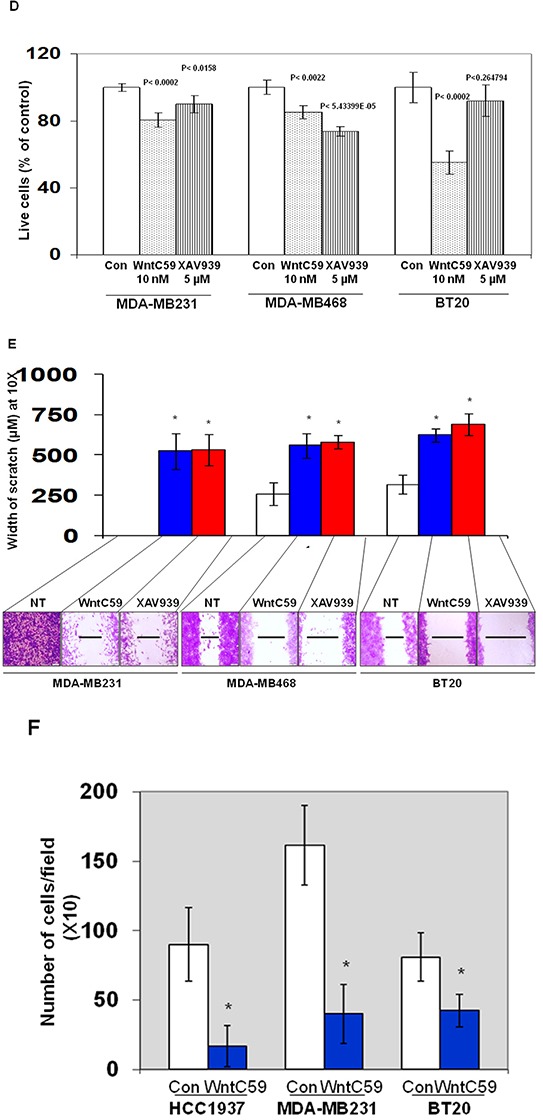
Effect of WP modulators on clonogenic growth (A-C), cell survival (D), fibronectin-directed migration (E) and matrigel invasion (F) in different TNBC cells **A.** WP activator, CHIR99021 upregulated while **B.** WP antagonists WntC59 and **C.** XAV939 downregulated 3D ON-TOP colony formation in MDA-MB231, SUM149, BT20 and MDA-MB468 cells. Two doses of WP activator CHIR99021 (3 μM and 6 μM), WP antagonists WntC59 (1 nM and 10 nM), and XAV939 (1 μM and 5 μM) were tested on the clonogenic growth of TNBC cells. Photomicrograph (X4 magnification) represents pictures of live cell colonies at the day 7 of treatment. **D.** Effect of Wnt-antagonists WntC59 (10 nM) and XAV939 (5 μM) on live MDA-MB231, MDA-MB468 and BT20 cells were tested by cell TiterGLO. The experiment was carried out in 10% FBS conditions. The effect was found to be more pronounced under 2.5 % FBS (data not shown). Statistical significance (p values) was presented on the respective bars. **E.** WP antagonists WntC59 (10 nM) and XAV939 (5 μM) blocked fibronectin-directed migration in MDA-MB231, MDA-MB468, BT20 cells (*p< 0.05). **F.** WntC59 (10 nM) downregulated matrigel invasion in HCC1937, MDA-MB231 and BT20 cells (*p< 0.05).

Different nodes of WP can be pharmacologically regulated [20, 34]. In this study, we used XAV939, an Axin1 and Axin2 stabilizing agent and WntC59, a compound that prevents palmitoylation of Wnt proteins by Porcupine, which thereby blocks Wnt ligand secretion and WP activity. Also, we have used an activator of WP. We used CHIR99021 to stimulate canonical Wnt signaling by inhibiting GSK3beta of the beta-catenin destruction complex. CHIR99021 is reported to induce a decrease in phosphorylation of beta-catenin and activation of the T-cell factor (TCF)-responsive TOP-Flash reporter, and recombinant Wnt3A has been known to replicate the effect of CHIR99021 [39]. Since we used LWnt3ACM for stimulating the WP (ligand-induced WP activation), we also decided to test the effect of the WP activator, CHIR99021 on MA phenotypes. Figure 8A showed that WP activator CHIR99021 enhanced the 3D colony formation in MDA-MB231, MDA-MB468 and BT20. This result is in contrast to the inhibitory effect of WP antagonists WntC59 (Figure 8B) and XAV939 (Figure 8C) on the clonogenic growth of MDA-MB231, MDA-MB468, SUM149 and BT20 TNBC cells. Figure 8D showed the effect of Wnt-antagonists WntC59 (10 nM) and XAV939 (5 microM) on live MDA-MB231, MDA-MB468 and BT20 cells by cell TiterGLO. The inhibitory effect of Wnt-antagonists was observed to be limited because of the presence of 10% FBS in the culture (to comply with the 3D clonogenic growth assay). A better inhibition of cell growth was however observed for XAV939 in cells with 2.5% FBS as reported by Bao et al. [40]. Wnt-antagonists WntC59 and XAV939 blocked the migration of MDA-MB231, MDA-MB468 and BT20 cells on fibronectin (Figure 8E) and significantly abrogated matrigel invasion in HCC1937, MDA-MB231 and BT20 cells (Figure 8F). Collectively, our data demonstrated that (1) WP activator CHIR99021, WP antagonist WntC59 and XAV939 regulated the clonogenic growth of TNBC cells in 3D ON-TOP colony formation assay (Figure 8A–8C), (2) WP antagonists WntC59 and XAV939 inhibited the growth of cells in cell TiterGLO assay (Figure 8D), (3) WP antagonists WntC59 and XAV939 blocked fibronectin-directed migration of cells in scratch assay (Figure 8E) and (4) WP antagonists WntC59 blocked fibronectin-directed matrigel-invasion of cells in transwell assay (Figure 8F).

### WP regulates metastasis-associated phenotypes in brain-metastasis specific TNBC cell line, MDA-MB231BR

**Figure 9:**
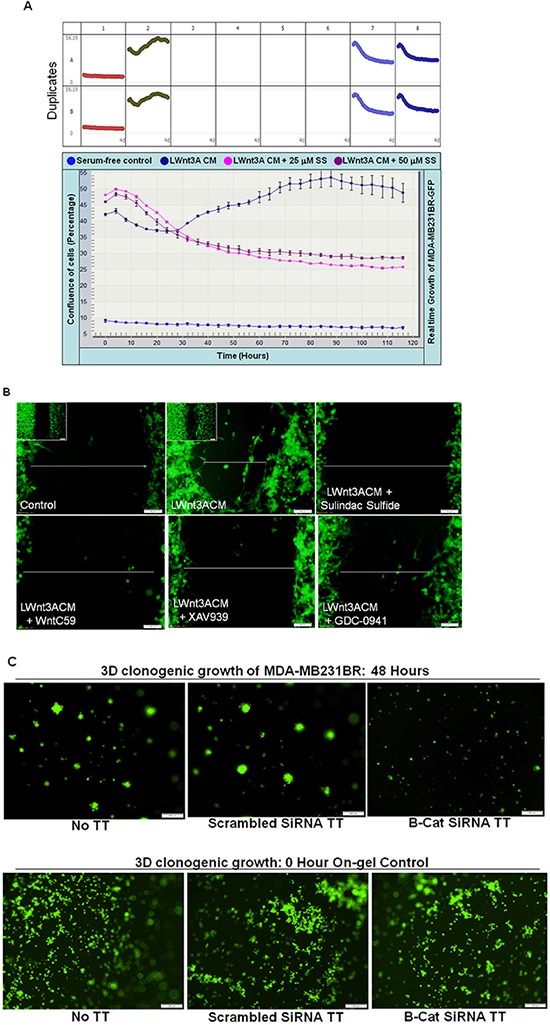
Effect of sulindac sulfide, WntC59, XAV939, GDC-0941 treatment and transient transfection of beta-catenin siRNA on real-time proliferation (A), fibronectin-directed migration (B) and clonogenic 3D growth (C) of brain metastasis-directed MDA-MB231BR TNBC cells MDA-MB231BR cells were used to test the role of the WP directly in real time proliferation **A.** fibronectin-directed migration **B.** and 3D clonogenic growth **C.** under different study conditions e.g. physiological stimulation of the WP by LWnt3ACM and genetic perturbation of beta-catenin following transient transfection of beta-catenin siRNA. (A) Sulindac sulfide blocked LWnt3ACM stimulated proliferation of cells as measured by the percentage of the confluence of cells (mean Vs time). (B) LWnt3ACM stimulated fibronectin-directed migration for 24 hours was abrogated in the presence of WP modulators, WntC59 10 nM as well as XAV939 (5 μM), sulindac sulfide (50 μM) and GDC-0941 (1 μM). **C.** LWnt3ACM stimulated 3D clonogenic growth for 48 hours was abrogated following transient transfection of beta-catenin siRNA as compared to the transfection of scrambled siRNA.

To test the specific effect of WP in the control of metastasis in TNBC, we used brain-metastasis specific TNBC cell line, MDA-MB231BR (courtesy Dr. P. S. Steeg). In line with the studies instituted by Dr. Steeg and her colleagues [41], our *in vivo* study showed that this cell line does metastasize in the brain of immune-compromised mice (data not shown). We tested the effect of sulindac sulfide on LWnt3ACM stimulated real-time growth of live MDA-MB231BR cells. Figure 9A showed that LWnt3ACM-stimulated real-time increase in the confluence (%) of MDA-MB231BR cells was blocked dose and time dependently by the treatment with sulindac sulfide as compared to serum free controls. We also used pharmacological modulators of different nodes of WP like WntC59, XAV939, sulindac sulfide and GDC-0941 (as tested in other TNBC cell lines in this study) to demonstrate a blockade of LWnt3ACM-stimulated fibronectin-directed migration in MDA-MB231BR cells (Figure 9B). Finally to prove the direct role of the WP in the metastasis (brain) in TNBC we tested the clonogenic growth of MDA-MB231BR cells following transfection of beta-catenin siRNA and demonstrated that beta-catenin siRNA-mediated perturbation of WP also abrogated LWnt3ACM-stimulated 3D clonogenic growth in MDA-MB231BR cells (Figure 9C). The figure showed that 3D clonogenic growth of cells for 48 hours was blocked in the absence of beta-catenin following 24 hours of siRNA transfection. Pictures of the “on-gel” control after 24 hours of siRNA transfection (lower panel) were presented for the reference.

## DISCUSSION

Metastatic progression is the single most important cause of poor outcomes in these TNBC patients. Tumor cells in the course of their evolution acquire genetic alteration(s) that triggers their metastatic potency via deregulation of different signaling pathways which singularly or collectively activates MA phenotypes. We have previously demonstrated that the upregulation of Wnt signaling in TNBC patients is associated with metastasis [20] which in agreement with reports from Reis-Filho's team and Khramtsov et al., clearly established that there is WP upregulation in TN subset of BC [19, 21]. This study is built on these reports which collectively demonstrate that the upregulation of Wnt signals is a cardinal feature of TNBC. Here we provide the mechanism based insight towards the understanding of the functional relevance of the WP in the biology of metastasizing TNBC cells. Our results demonstrated that TNBC cells acquire migratory, invasive and clonogenic properties via upregulation of WP and thus WP upregulation observed in tumors of TNBC patients as we reported earlier is functionally associated with MA phenotypes. Our data mechanistically explained how the upregulation of different components of the WP are causally associated with metastasis and poor prognosis in this subtype of BC as reported earlier [20].

TNBC is a heterogeneous disease [8] with different genome based sub-sets within the subtype. This heterogeneity had been known to limit the outcome of the disease by posing real challenges for identifying targets and detecting driving pathways [42] as well as in translating TNBC gene signatures into clinics [43]. Recently Martínez-Revollar et al., have reported that heterogeneity between TNBC cells is due to a differential activation of Wnt and PI3K/AKT pathways [44]. To address the inherent genetic heterogeneity of TNBC, we chose 15 different TNBC cell lines with a wide range of genetic backgrounds representing different genetic alterations in TNBC including *p53* mutation, *BRCA1* mutation, *BRCA2* mutation, PTEN-nullness, *PIK3CA* mutation, *RB1*-mutation, *RAS-RAF* mutation as reported earlier [20, 24]. This helped us to model all major characteristic genetic alterations observed in TNBC. Our study included TNBC cell lines representing all major types of genetic alterations as mentioned in detail in the method section. We have used (1) physiological (stimulation by LWnt3A Condition Media; LWnt3ACM), (2) genetic (beta-catenin siRNA), (3) pharmacological (WP activator CHIR99021, WP antagonist WntC59, WP antagonist XAV939 and PI3K pathway inhibitor GDC-0941) and (4) mechanistic (WP signaling inhibitor, sulindac sulfide) methods to understand the functional relationship between WP and MA phenotypes in TNBC. In summary, our study involved a comprehensive testing of different modulators of WP on different nodes of WP in the context of different MA phenotypes in TNBC cells representing all major genetic-alteration-based subsets. We observed that cell lines with different genetic background did have a different response to different agents in the context of different phenotypes. For example, we observed that *RAS/RAF* mutated mesenchymal TNBC cell line MDA-MB231 was most effective in migrating on fibronectin as compared to PTEN null epithelial MDA-MB468 cells. This effect was reciprocated in fibronectin-dependent matrigel invasion.

**Figure 10:**
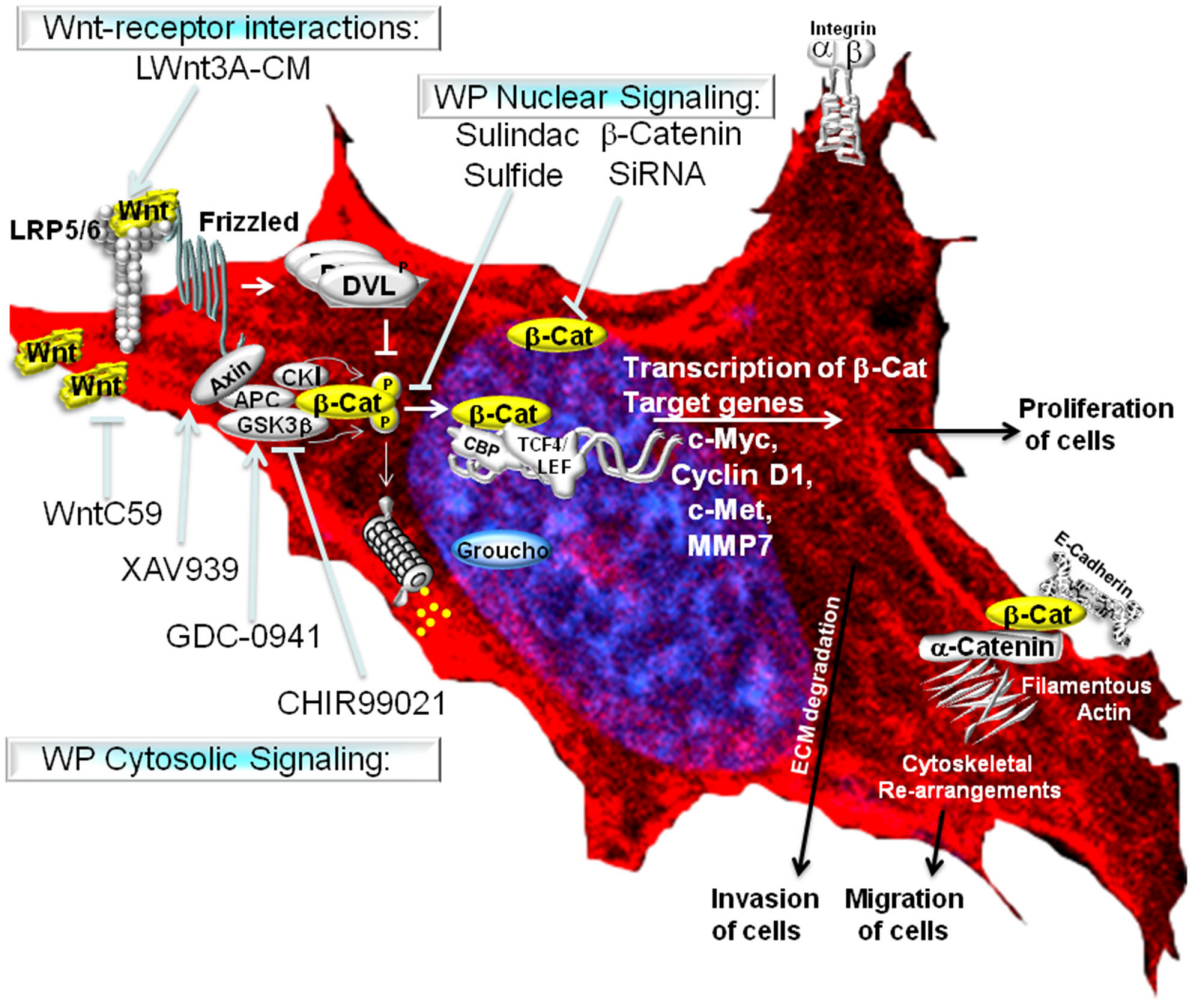
WP controls metastasis-associated phenotypes in TNBC The Wnt signaling pathway is activated following the binding of extracellular Wnt ligands (often secreted by the cell) to Frizzled transmembrane receptors. Canonical Wnt signaling causes the activation of beta-catenin-TCF complexes leading to the transcriptional activation of beta-catenin target genes (as stated in the discussion).

In our study, we have used physiological, pharmacological and genetic as well as classical cell signaling methods to stimulate and block WP, and studied the role of WP in the regulation of MA phenotypes in TNBC. The upregulation of WP leads to an enhanced transcriptional activity of beta-catenin via a canonical pathway in TNBC cells (Figure 10). In the absence of WNT signals, the rapid turnover of newly synthesized beta-catenin is orchestrated via sequestration of free cytoplasmic beta-catenin by the ‘scaffolding/destruction’ complex, consisting of Axin, APC, CK1 and GSK3beta where beta-catenin is phosphorylated for its subsequent proteasomal degradation. Binding of WNT to Frizzled triggers the recruitment (and phosphorylation) of Dishevelled (DVL) and Axin by Frizzled and the WNT co-receptor LRP respectively. GSK3beta is released from the ‘scaffolding/destruction’ complex. As a result, unphosphorylated and transcriptionally active beta-catenin moves to the nucleus where it interacts with members of the TCF and LEF family of transcription factors to induce transcription of beta-catenin's downstream target genes including *c-MYC, MMP7, cyclin D1* and *c-Met* (http://www.stanford.edu/~rnusse/pathways/targets.html). TCF and LEF proteins act as transcriptional repressors by binding to Groucho proteins in the absence of beta-catenin (Figure 10). Our primary validation strategy was focused on beta-catenin because beta-catenin is the functional readout of WP that mediates Wnt signaling [28]. We have used pharmacological and genetic tools to attenuate cellular levels of beta-catenin. Since the activation of the Wnt-beta-catenin pathway leads to an increase of beta-catenin protein via inhibition of its degradation [45] and sulindac sulfide mediated loss of beta-catenin levels occurs via reactivation of proteasomal degradation [31], we chose sulindac sulfide for the attenuation of the pathway in order to test our hypothesis (Figure 10). Matsuda et al., inhibited the Wnt-beta-catenin pathway following ectopic expression of sFRP1 in MDA-MB231 BC cells as determined by the levels of total beta-catenin [46]. Similarly, in our study, total beta-catenin was decreased following the administration of sulindac sulfide. Abnormal migration of tumor cells following specific integrin engagement and invasion of tumor cells through ECM are among the key phenotypic components of metastasis. Since 1) directional migration of tumor cells is one of the critical prerequisites for the metastatic behavior of TNBC and 2) Wnt-beta-catenin signaling is known to control migration of tumor cells, we studied the effect of inhibition of the WP by sulindac sulfide on fibronectin-directed migration for 24 hours in TNBC cell lines. A similar role of beta-catenin in cell migration has been demonstrated during epithelial tubulogenesis wherein the expression of *D*N90 and *D*N131 beta-catenin was found to inhibit directed cell extension and migration from MDCK cysts [47]. We verified our results in real-time, by video microscopic recording the movement of live TNBC cells on fibronectin (supplementary movie; M1 & M2) and semi-quantifying the movement of the cells (from the image analyses of the Time-lapsed images) following sulindac sulfide (50 microM) administration in scratch-wound healing assay (Figure 2E). Beta-catenin is a multifunctional protein that performs many functions in a cell including E-cadherin-mediated adhesion, and in membrane extensions. Intracellularly E-cadherin is linked to catenins (alpha-, beta-, and gamma-catenin/plakoglobin), which connect E-cadherin to the actin cytoskeleton [48]. Inhibitory effect of sulindac sulfide on fibronectin-directed migration was in agreement with the alterations of the cytoarchitecture of filamentous actin (Figure 3) and podia characteristics of TNBC cells (Figure 4). Beta-catenin forms an adhesion complex with E-cadherins, alpha-catenin as well as actin and this adhesion complex is important for the membrane translocation of beta-catenin [28]. Interestingly, we observed an inverse correlation between E-cadherin expression and fibronectin-mediated migration in different TNBC cells (Supplementary Figure S3). TNBC cells expressing comparatively low levels of E-cadherin (HCC1937, MDA-MB231, MDA-MB468, and Hs578t) exhibit higher migratory responses as compared to the high E-cadherin-expressing TNBC cells (HCC70 and BT20) (Supplementary Figure S3). Our observed differential expression of E-cadherin in TNBC cell lines is in agreement with Sommers, et al., [49] who also reported a lack of expression of this protein in MDA-MB231, MDA-MB436, BT549, and Hs578t. E-cadherin has been termed an “invasion suppressor” while activated beta-catenin stimulates motility of epithelial cells [50]. Participation of E-cadherin in the WP was suggested because of the dual role of beta-catenin in cell adhesion and the Wnt signaling pathway. Beta-catenin interacts at the cell membrane with the cell adhesion protein E-cadherin while it activates Wnt target genes in the nucleus. The intracellular domain of E-cadherin interacts with the actin cytoskeleton via a protein complex containing alpha-catenin, beta-catenin, and gamma-catenin [51]. Thus, the inverse relationship between E-cadherin expression and TNBC cell migration observed in our results may substantiate the fact that the existing role of WP in TNBC is functionally connected to the actin cytoskeleton. In the light of above discussion, our data raise the obvious question regarding the role of canonical and non-canonical WP in mediating MA phenotypes in TNBC.

A direct involvement of WP in the regulation of MA phenotypes, fibronectin-directed migration, invasion and 3D-clonogenic growth was inferred from the result of both perturbation of WP and stimulation of WP that was carried out finally in the brain metastasis-specific MDA-MB231BR TNBC cells. Our data collectively provided a direct causal relationship between WP upregulation and metastasis in TNBC cells. Considering the clinical impact of the aggressive nature of TNBC on the outcome in patients, our data will be worthwhile to consider for establishing biomarkers in this subtype of BC especially in the metastatic setting. Interestingly, in an article of November 2015 Zhang et al., reported that PTEN loss primes brain metastasis outgrowth in subtypes of BC. Their study demonstrated that an adaptive PTEN loss in brain metastatic tumor cells led to a reciprocal enhancement of the outgrowth of brain metastatic tumor cells via enhanced proliferation and reduced apoptosis [52]. Dysregulated PI3K/AKT and WNT/CTNNB1 signaling in the development and progression of metastatic tumor is mediated via a direct inhibition of beta-catenin degradation following the inhibition of GSK3beta. LWnt3ACM-stimulated integrin-directed migration of brain metastasis-specific MDA-MB231BR cells was inhibited following GDC-0941 treatment (Figure 9B) in our study. Also an inhibitor of GSK3beta, CHIR99021 has been shown to enhance the 3D clonogenic growth of different TNBC cells including BT20, which expresses the WNT3 and the WNT7B oncogenes (Figure 8A). We have previously identified that the functional upregulation of secreted-MMP7, a transcriptional target of WP in TNBC is associated with the loss of PTEN [24], a tumor suppressor gene whose loss has been found to be the most common first event associated with basal-like subtype [53] and this PI3K-AKT pathway activating event due to a deletion/mutation/loss of PTEN frequently (35%) occurs in TNBC [54]. A synergistic effect of PTEN loss and WNT/CTNNB1 signaling pathway activation has been reported in tumor development and progression [55]. Thus, it is possible that an adaptive loss of PTEN may be functionally responsible for the upregulation of WP during the process of metastatic progression of triple negative breast tumor cells in the brain. It remains to identify however the exact functional relationship between the adaptive loss of PTEN in TNBC tumor cells those are destined for brain metastasis and the upregulation of WP. Recently various unconventional and novel agents have been tested experimentally for their ability to suppress metastatic TNBC including models of metastases to the brain [56]. Our data provides first experimental evidence to consider the role of the WP as an effective new target for the future development of anti-metastasis therapies in TNBC, especially in the context of brain metastasis and highlights the importance of the WP in the search of novel treatment as well as identification of collective biomarkers in metastatic TNBC.

## MATERIALS AND METHODS

### Data analyses using cBioPortal

We studied alterations (amplification, deep deletion, missense mutation, mRNA upregulation, mRNA downregulation, protein upregulation and protein downregulation) in *CTNNB1*, *APC* and *DVL1* genes in Breast Invasive Carcinoma (TCGA, 2012) case set and Invasive Ductal Cancers (brca/tcga/pub2015) case set using c-BioPortal (figure 1 & S5). The genomic study selected were (1) missense mutations, (2) amplifications (3) deep deletion (4) putative copy-number alteration from GISTIC, (5) mRNA expression Z-scores (microarray) with Z-score thresholds ± 2.0 and (6) protein/phosphoprotein levels (RPPA) with Z-score thresholds ± 2.0. In Breast Invasive Carcinoma (TCGA, 2012) case set, we have selected (1) all tumor samples (825 patients / 825 samples) and (2) PAM50 Basal subtype (81 patients/81 samples). In Invasive Ductal Cancer (brca/tcga/pub2015) case set, we have selected (1) all tumor samples (1105 cases/patients), (2) Invasive Ductal Cancer, Luminal A (201 cases/patients), (3) Invasive Ductal Cancer, PAM50 Luminal B (122 cases/patients) (4) Invasive Ductal Cancer, PAM50 Her2-enriched (51 cases/patients), (5) Invasive Ductal Cancer, PAM50 Basal-like (107 cases/patients) and (6) Triple-negative breast tumors (82 cases/patients). Advanced cancer genomic data visualization is obtained with the help of “The Onco Query Language (OQL)”. OncoPrints represents compact means of visualizing distinctive genomic alterations, including somatic mutations, copy number variations (CNV), and mRNA expression changes occurring across a set of cases. We used the Onco Query Language (OQL) to select and define genetic alterations for all the OncoPrint outputs on the cBioPortal for Cancer Genomics. Oncoprints (different levels of zoom) have been generated using cBioPortal. OncoPrints are used for visualizing gene set as well as pathway alterations across a set of cases, and for visually identifying trends, such as trends in mutual exclusivity or co-occurrence between gene pairs within a gene set. Tumor types (tumor data sets) are chosen in accordance with the publication guidelines (as updated on January 17^th^, 2014) of TCGA (tcga@mail.nih.gov). cBioPortal data is subjected to scheduled updates. We acknowledge the cBioPortal for Cancer Genomics site (http://cbioportal.org) which provides a web resource for exploring, visualizing, and analyzing multidimensional cancer genomics data.

Cell lines, Reagents Drugs and Antibodies: TNBC cell lines (HCC38, HCC70, HCC1143, HCC1187, HCC1008, HCC1937, MDA-MB231, MDA-MB468, BT20, BT549, Hs578t, MDA-MB157, MDA-MB436, SUM102 and SUM149), hormone receptor-positive cell line (MCF-7), and HER2-positive cell lines (BT474, BTH474, and SKBr3) were cultured according to a standard protocol as mentioned earlier [24]. Our study included TNBC cell lines representing all major types of genetic alterations as mentioned in detail in the discussion section. We have included PTEN-null (MDA-MB468), *CDKN2A*-alterations (BT20, HCC38, MDA-MB231), *RAS/RAF* mutated (MDA-MB231), *RB1*-alteration (MDA-MB468), *BRCA*-competent (MDA-MB231, MDA-MB468), *BRCA*-incompetent (HCC1937, SUM149), EGFR-overexpressed (MDA-MB468), *p53*-mutated (HCC1187, BT20, HCC70, HCC1937, HCC38, MDA-MB231, MDA-MB468) and *PIK3CA*-mutated (BT20) as well as histology like IDC (SUM149), adenocarcinoma (MDA-MB231, MDA-MB468), Carcinoma, primary ductal (HCC38, HCC70) in our study. TNBC cell line BT20 which expresses the WNT3 and the WNT7B oncogenes were used [25]. To test a direct involvement of WP in metastasis in TNBC, we used EGFP-tagged brain metastasis-specific cell line, MDA-MB231BR (A kind gift from Dr. P. S. Steeg; Women's Malignancies Branch, Center for Cancer Research, National Cancer Institute, NIH, Bethesda, MD, USA). All BC cell lines except SUM102, SUM149 and Herceptin (trastuzumab) resistant BT474HER (BTH) cell lines were obtained from ATCC. SUM102 and SUM149 cells were obtained from Asterand (Partners in human tissue research). Herceptin resistant BT474 cell was obtained from Dr. Mark Pegram (Div. Hemat. & Med. Oncology, UCLA, CA). Antibodies including tubulin (BD Biosciences, CA), actin, c-MYC, beta-catenin, and MMP7 (Abcam Inc., Cambridge, MA) were used for the study. WP modulators, CHIR99021, WntC59 and XAV939 were procured from Cellagen Technology.

GDC-0941 was procured from selleckchem.com. Sulindac sulfide was obtained from Sigma-Aldrich.

### Biochemical analysis

We performed immunoblots by Western blots on the equivalent amounts of protein (Bradford assay) using clarified cell lysates as mentioned earlier [26]. Cell lysates were assayed for total protein using BSA as standard. Normalized clarified lysates (25-50 μg protein) were resolved by 10% SDS-PAGE. Membranes are immunoblotted with different primary antibodies before individual bands on nitrocellulose membranes were visualized by ECL (Amersham Pharmacia Biotech, UK) using UVP.

### Integrin-directed migration and invasion assays

Haptotaxis and wound healing assays were performed to test fibronectin-directed migration of tumor cells. Haptotaxis and matrigel-invasion were carried out using transwell migration chambers. An adhesion assay on fibronectin was performed simultaneously with the haptotaxis assay under similar conditions. *In vitro* wound healing migration assays were performed (scratch assay) as previously described by our group [20] [24] [26].

### Actin dynamics and podia-parameters

TNBC cells were plated on fibronectin and treated with sulindac sulfide under conditions similar to that of the migration experiments. Cells were processed for Phalloidin-555 staining for filamentous actin. Nuclei were counterstained with DAPI. Cells were photographed using a Zeiss (Thornwood, NY) LSM 510 Meta confocal microscope with a 63 x (1.4-numerical-aperture) or 40 x (1.4-numerical-aperture) Plan-Apochromat oil objective as mentioned earlier [24]. Lamellipodia/cell and filopodia/cell were identified under a confocal microscope. For the semi-quantification purpose, an equal number of cells from the control and treated experiments exhibiting, at least, one filopodia were chosen from randomly selected fields, and their screenshot images were collected. A total number of lamellipodia/cell and filopodia/cell from the control, as well as treated groups, were counted manually from these screenshot confocal images.

### Real-time migration of live TNBC cells

Digitized bright-field time-lapse real-time images of the movement of MDA-MB231 cells (placed in live-cell imaging chambers) into the scratched area (for migration assay) were acquired with a Perkin Elmer Ultraview ERS (Norwalk, CT) disk-spinning confocal system, mounted on a Zeiss Axiovert 200M inverted microscope. To account for the axial focal changes of cells as they move, optical Z-sections were collected at 0.95 μm interval spacing (Movies: Supplementary data M1 & M2).

### Motility descriptors of real-time movement of live TNBC cells

Image analysis was carried out to measure the motility from the time-lapse images of the effect of sulindac sulfide on the fibronectin-directed migration of the TNBC cells in the scratch-wound assay on fibronectin. Nuclear tracking paths of migrating cells were shown as track overlays. The trajectory of the movement of cells is characterized by two quantitative motility descriptors (average velocity and MRDO, maximum relative distance from the origin). The position of the nucleus is taken as the point of “origin”. Time-lapse images are acquired with a Perkin Elmer Ultraview ERS confocal system. Bright-field images are acquired with a Hamamatsu Orca-ER camera (10x objective) at 10 minutes intervals.

### Casein zymography

Enzymatic activity of the secreted MMP7 from the conditioned media was determined by zymography using Bio-Rad precast casein gels as described earlier [27].

### Cell survival and proliferation assay

The cell survival assay was performed using cell Titer-GLO luminescent cell viability assay kit as per manufacturer's direction (Promega). The real-time proliferation of GFP positive MDA-MB231BR cells was performed using time-lapse imaging without labels (IncuCyte; ESSEN BioScience). Using live cells we generated long-term growth and growth inhibition curves and monitored morphology. The time course of the percentage of the confluence of LWnt3ACM stimulated GFP positive MDA-MB231BR cells (Mean Vs time) was represented by four days following two doses of sulindac sulfide (25 μM and 50 μM).

### Clonogenic 3D ON-TOP assay

Appropriate three-dimensional (3D) culture provides a more physiologically relevant approach to the analysis of gene function and cell phenotype *ex vivo*. We tested the involvement of WP in 3D ON-TOP cultures of the laminin-rich extracellular matrix as many of the crucial micro-environmental cues are restored using this form of culture as previously described [26].

### LWnt3A conditioned media

LWnt3A conditioned medium was obtained from the LWnt-3A (ATCC® CRL-2647™) cells. These L-M(TK-) cells were transfected with a Wnt-3A expression vector and stable clones were selected in medium containing G418. The *Wnt3A* gene encodes a secreted glycoprotein. The cells are engineered to secrete biologically active Wnt3A protein. We used these cells as our source for production of Wnt3A conditioned medium (Wnt3ACM) (as described by the ATCC). L Cell (ATCC® CRL2648™) was used as the parental line for the LWnt3A cell line.

### LWnt3ACM stimulation following transient transfection of beta-catenin siRNA

The transient transfection of beta-catenin siRNA was performed as reported before [27]. GFP-positive MDA-MB231BR cells were either not transfected or transiently transfected with scrambled siRNA or beta-catenin siRNA for 24 hours. After 24 hours, cells were plated (zero hours on-gel control) for 3D clonogenic growth for 48 hours. Pictures of zero hours on-gel control for not transfected scrambled siRNA transfected and beta-catenin siRNA transfected MDA-MB231BR cells were presented for the reference. Pictures of live cells in 3D culture were taken using Olympus DP72 microscope. Since the LWnt3ACM was obtained under FBS-free conditions, the control in these experiments represents FBS-free controls. For the clonogenic study, cells were transiently transfected with beta-catenin siRNA. Following 24 hours of transfection cells were plated for the 2D and 3D clonogenic assays.

### Statistical analysis

Each experiment was independently repeated 4-5 times. The Student t-test was used to evaluate differences observed between treated groups and vehicle treated controls for other experiments. Data presented in the graphs represent the Mean ± S.D. of results. The minimum level of statistical significance was set at p<0.05. Inter-group comparison was made with a paired two-sample (Student t-test). Phenotypic experiments are performed in quadruplets.

## SUPPLEMENTARY FIGURES AND MOVIES






