# Supplementary material for: Wnt-beta-catenin pathway signals metastasis-associated tumor cell phenotypes in triple negative breast cancers
Source: Oncotarget. 2016 Apr 26;7(28):43124–49. doi: 10.18632/oncotarget.8988 (PMC5190013; doi:10.18632/oncotarget.8988)
Supplement: Supplementary file 1 [file oncotarget-07-43124-s001.pdf]

Wnt-beta-catenin pathway signals metastasis-associated tumor cell phenotypes in triple negative breast cancers

SUPPLEMENTARY FIGURES AND MOVIES

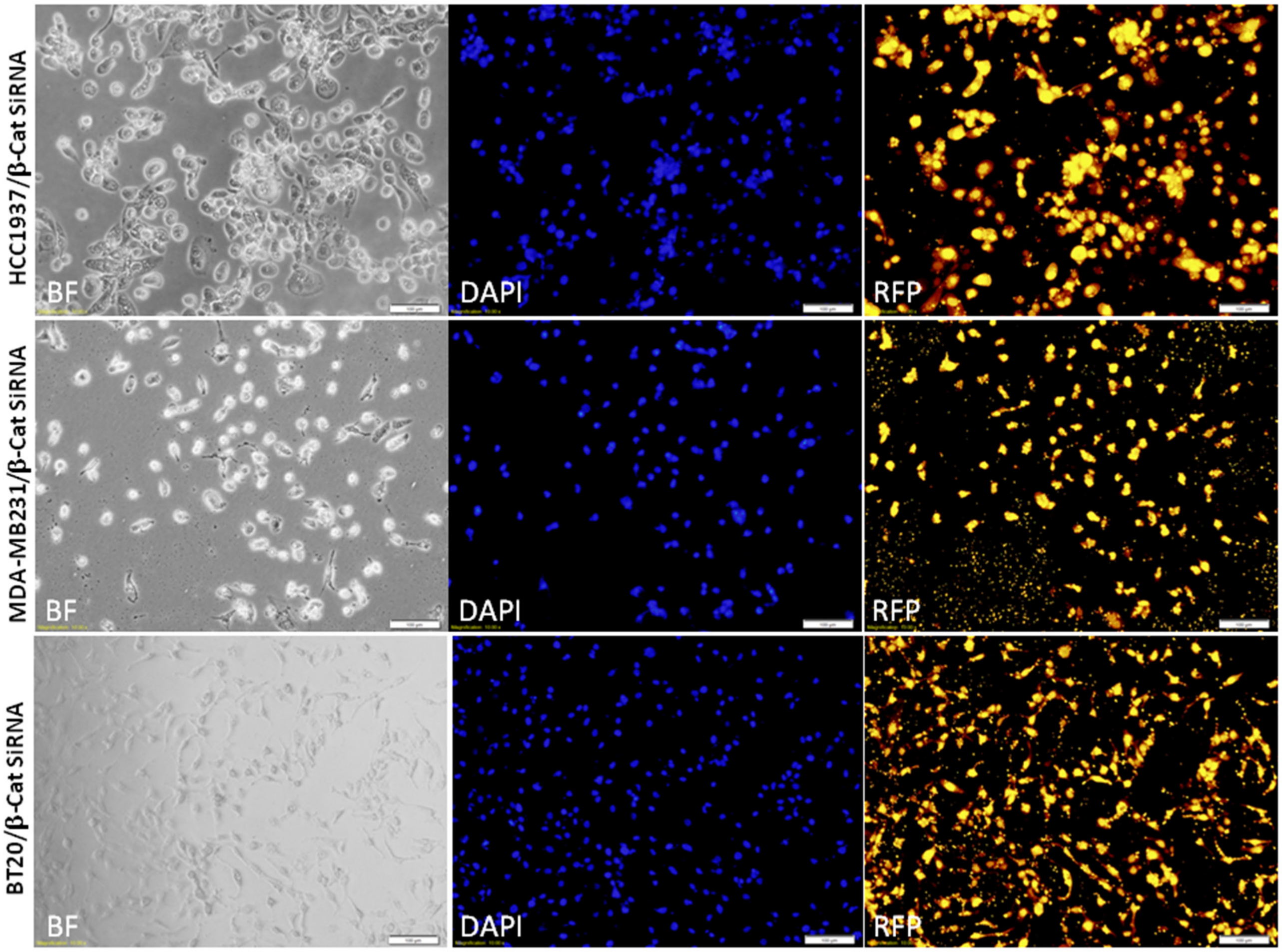

Supplementary Figure S1: Transient transfection efficiency of beta-catenin siRNA in TNBC cells was tested using RFP. Bright field and DAPI stained pictures are included for the same field as the reference.

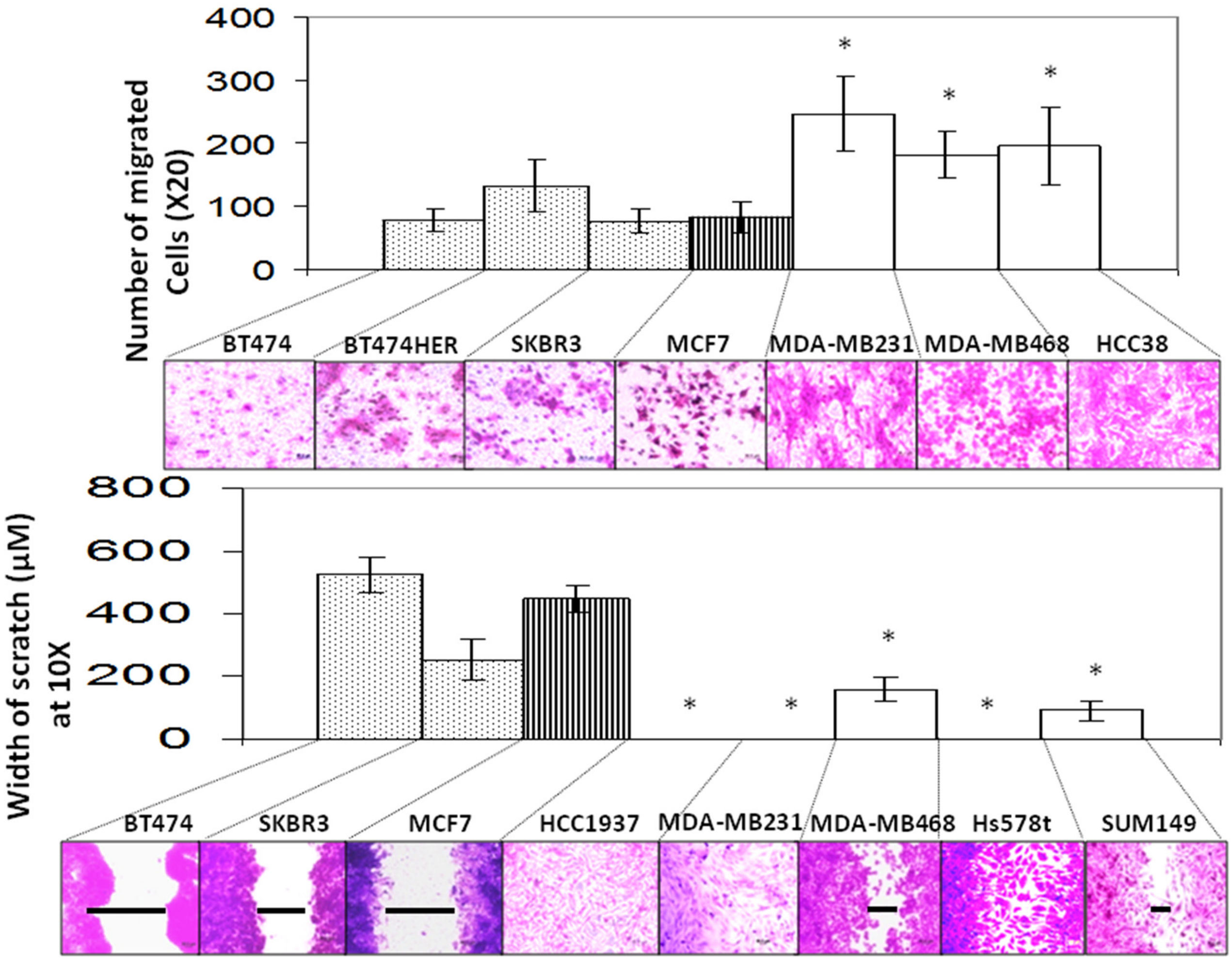

Supplementary Figure S2: Fibronectin-directed migration in TNBC, HR and HER2+ BC cell lines using both transwell and scratch assays are presented. Upper panel shows the fibronectin-mediated migration of HR+ MCF7 cells, HER2-overexpressed BT474, BTH474 and SKBr3 cells, and MDA-MB231, MDA-MB468, HCC38 TNBC cell lines by transwell assay for 24 hours. Each bar represents Mean ± SD of the number of cells migrated. n=8-10, *p< 0.05. The lower panel shows the fibronectin-mediated migration of HR+ MCF7 cells, HER2-overexpressed BT474, BTH474 and SKBr3 cells, and MDA-MB231, MDA-MB468, HCC38 TNBC cell lines by scratch assay for 24 hours. Each bar represents Mean ± SD of the width of the scratch. The black line indicates distance measured in micron). n=8-10, *p< 0.05.

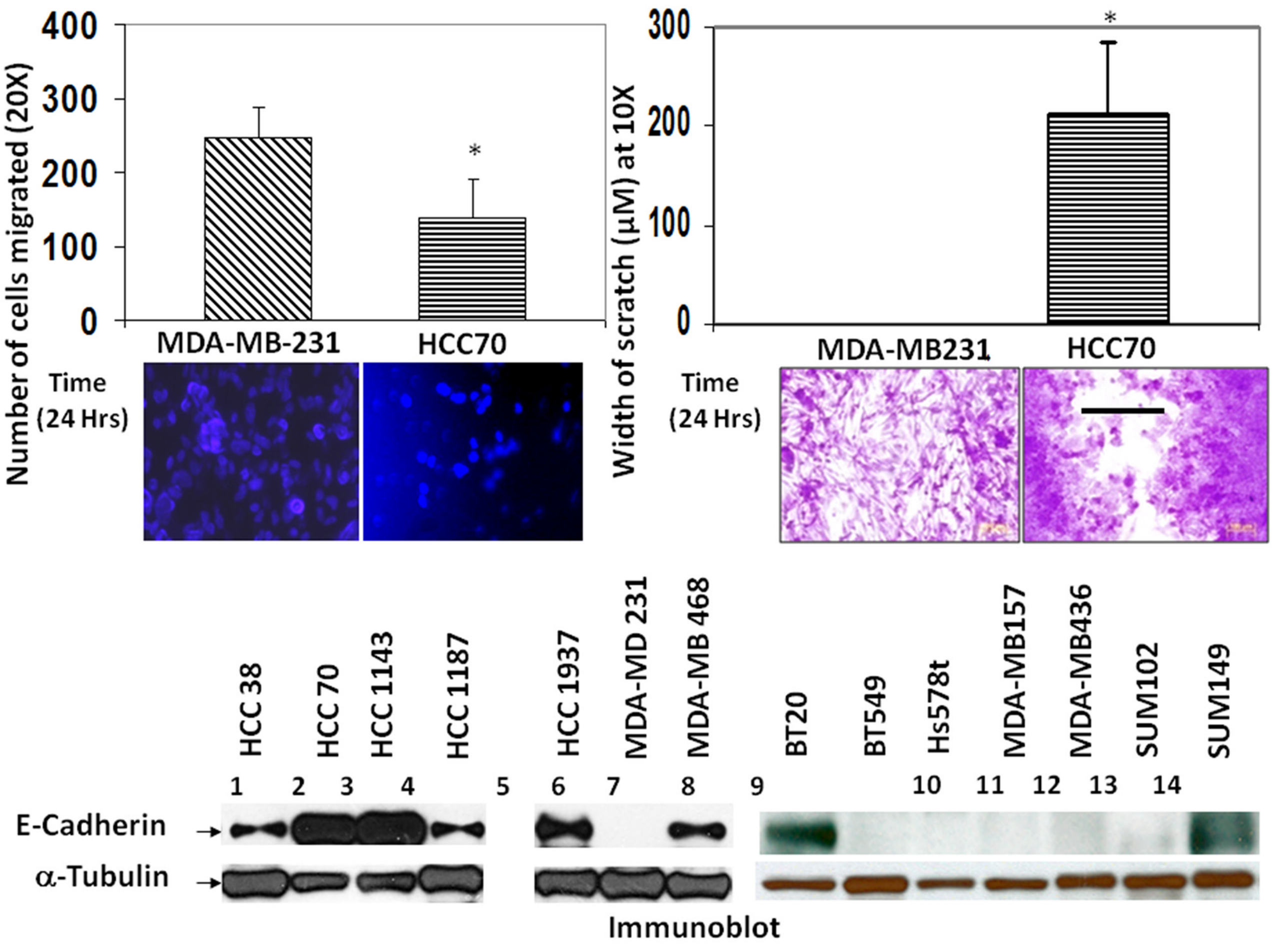

Supplementary Figure S3: Correlation between the expression levels of E-cadherin and fibronectin directed migration in TNBC cell lines: E-cadherin expression (lower panel) and fibronectin-directed migration (Transwell assay and Scratch Assay) in HCC70 and MDA-MB231 (upper panel) TNBC cell lines for 24 hours are presented (*p< 0.05). Immunoblot (lower panel) shows higher expression of E-cadherin (lanes 2, 3, 8, 14) as compared to (lanes 1, 4, 5, 6, 7, 9-13) in TNBC cell lines. Alpha-tubulin expression is used as loading control.

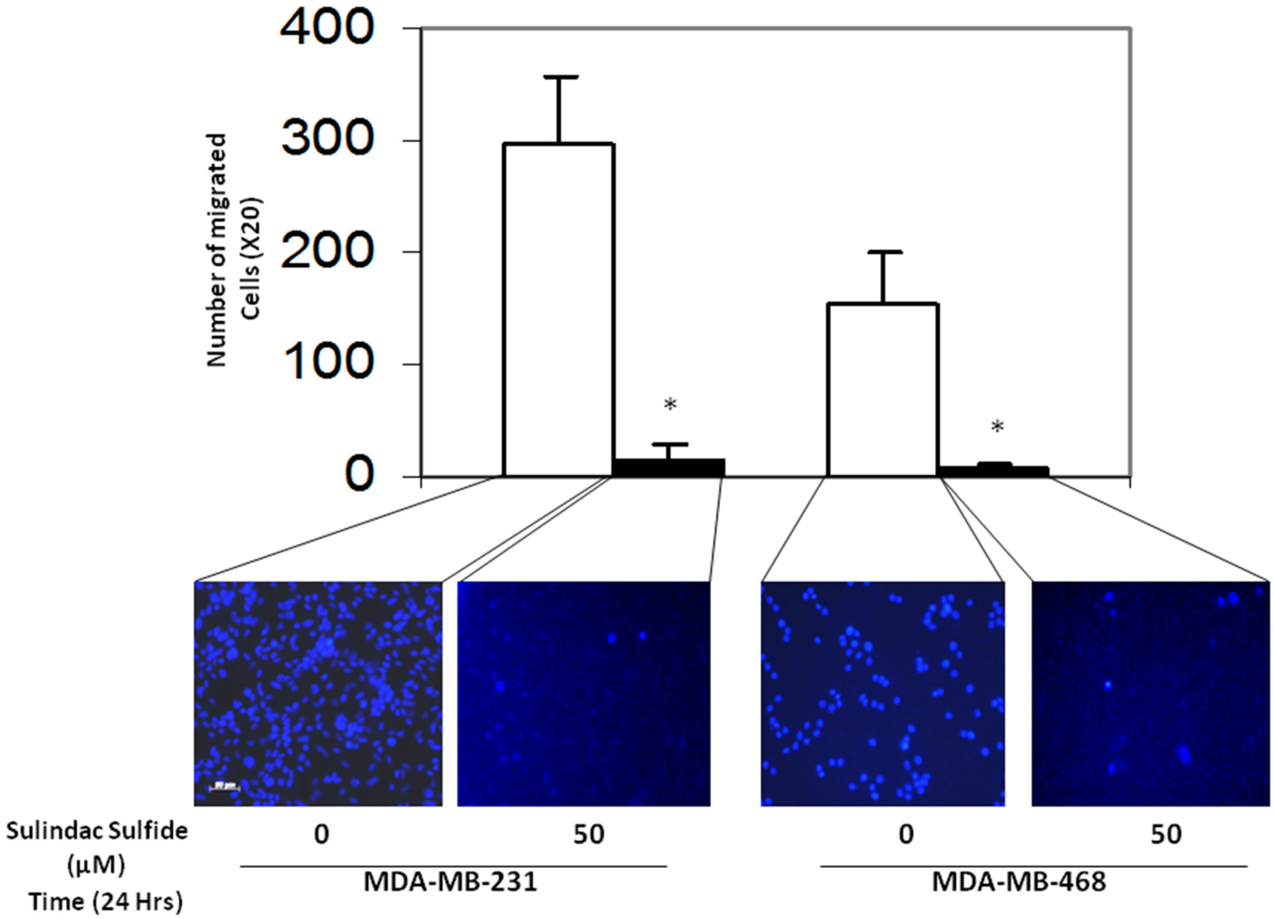

Supplementary Figure S4: Sulindac sulfide blocked fibronectin directed migration of TNBC cell lines in transwell assay for 24 hours. Each bar represents Mean ± SD of the number of cells migrated as counted in randomly chosen fields following DAPI staining. n=8-10, *p< 0.05.

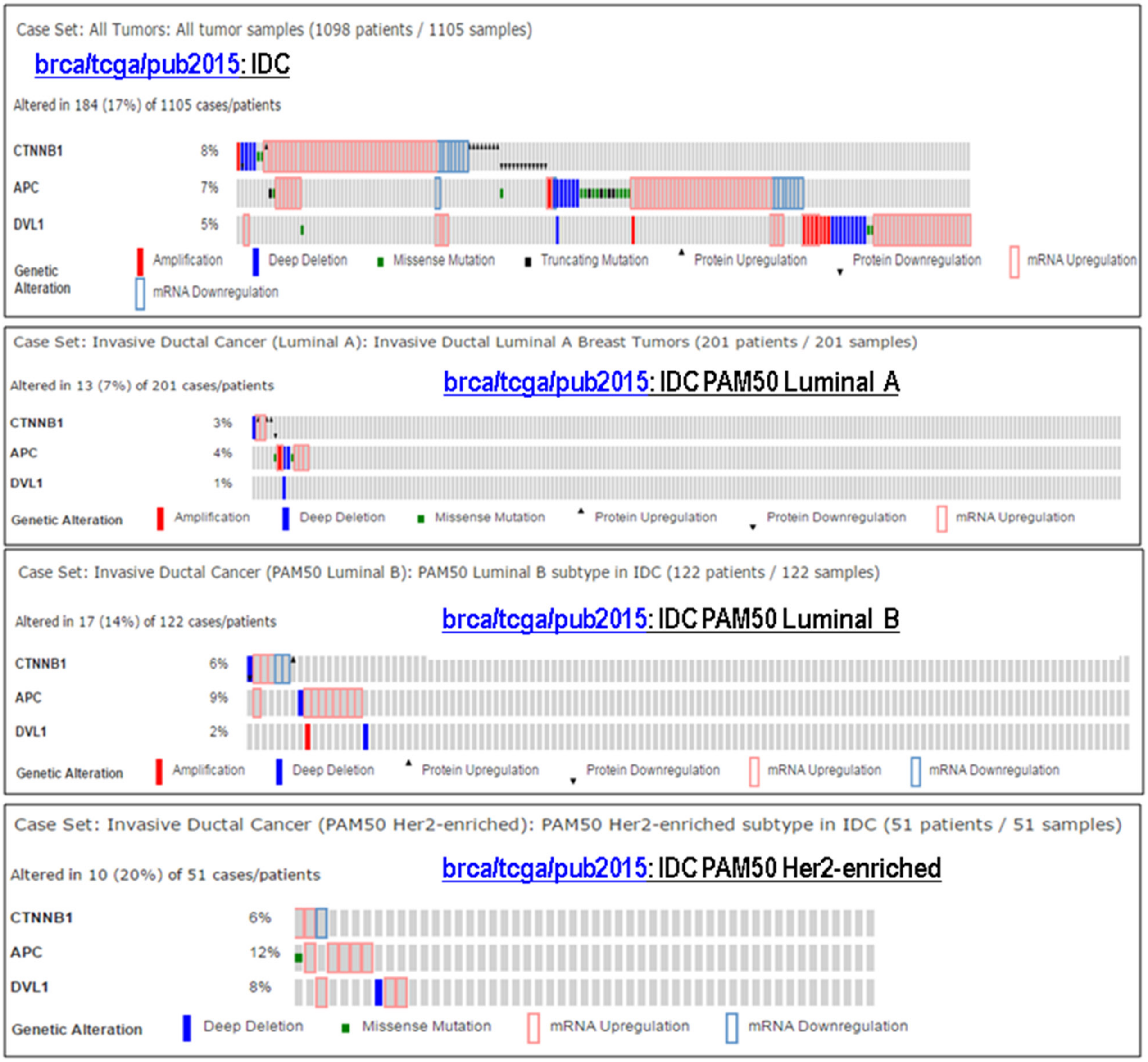

Supplementary Figure S5: Alterations of WP genes in BC and its subtypes: Oncoprints showing alterations in WP associated three genes *CTNNB1, APC and DVL1* (amplification, deep deletion, missense mutation, mRNA upregulation, mRNA downregulation, protein upregulation and protein downregulation) in IDC, brca/tcga/pub2015 (uppermost panel), IDC PAM50 Luminal A brca/tcga/pub2015 (upper middle panel), IDC PAM50 Luminal B brca/tcga/pub2015 (lower middle panel) and IDC PAM50 Her2-enriched brca/tcga/pub2015 (lowermost panel). The oncoprints are generated using 1098 patients/1105 samples for all tumors, using 201 patients/201 samples for IDC PAM50 Luminal A, using 122 patients/122 samples for IDC PAM50 Luminal B and using 51 patients/51 samples for IDC PAM50 Her2-enriched. Oncoprints (different levels of zoom) have been generated using cBioPortal. Protein level obtained from IHC staining (cBioPortal).

M1

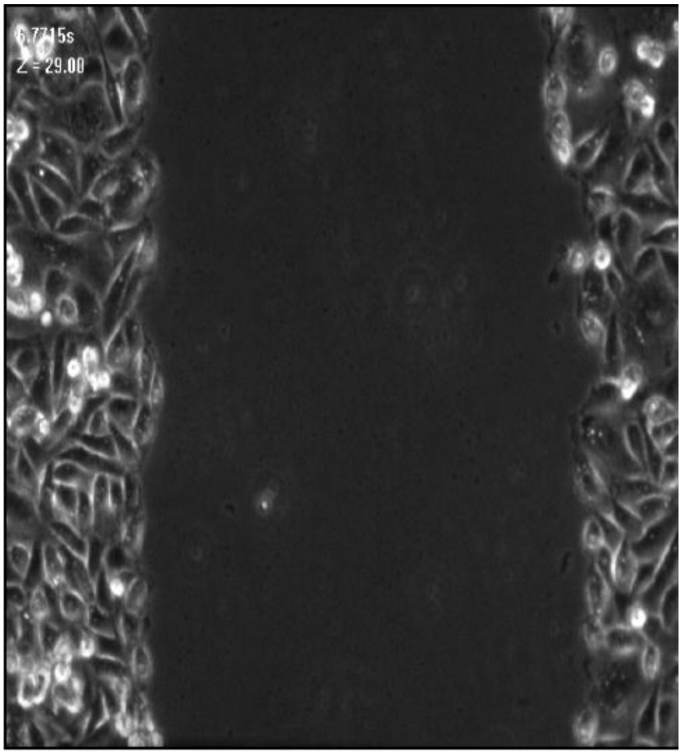

M2

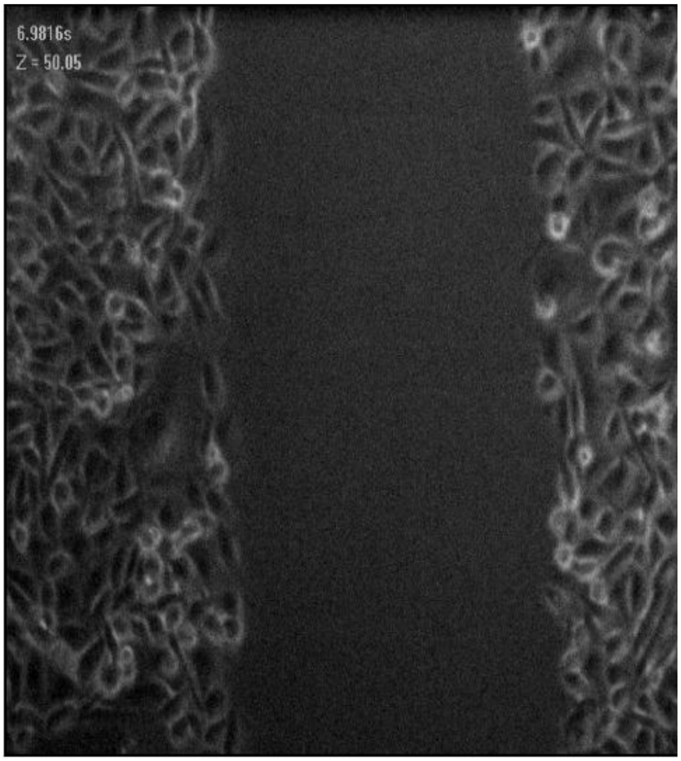

Supplementary Movie M1 & M2: Real-time migration of MDA-MB231TNBC cell on fibronectin (M1) and its inhibition by sulindac sulfide (M2): Sulindac sulfide (50 µM) blocked fibronectin-directed real-time migration of live MDA-MB231 cell line in the wound healing scratch assay.
